# Treatment outcomes for adolescent bulimia nervosa: a systematic scoping review of quantitative findings

**DOI:** 10.1186/s40337-025-01236-8

**Published:** 2025-04-16

**Authors:** Madeleine Love, Julian Baudinet

**Affiliations:** 1 Institute of Psychiatry, Psychology and Neuroscience, Kings College London, 16 De Crespigny Park, London, SE5 8AB UK; 2 Centre for Research in Eating and Weight Disorders (CREW), Institute of Psychiatry, Psychology and Neuroscience, Kings College London, 16 De Crespigny Park, London, SE5 8AB UK; 3 Maudsley Centre for Child and Adolescent Eating Disorders (MCCAED), South London and Maudsley NHS Foundation Trust, De Crespigny Park, Denmark Hill, London, SE5 8AZ UK

**Keywords:** Adolescent, Child, Bulimia nervosa, Eating disorder not otherwise specified, EDNOS, Treatment, Family-based treatment, CBT

## Abstract

**Background:**

This study aimed to systematically scope the available quantitative evidence for adolescent Bulimia Nervosa (BN) interventions. Specifically, the study aimed to review psychological and behavioural symptoms outcomes, as well as changes in comorbid psychiatric and caregiver factors.

**Method:**

Five main and three grey literature databases were searched on 4th September 2024. Eligible peer-reviewed journal articles, dissertations and book chapters were included. Studies included children and adolescents with primary diagnoses of Bulimia Nervosa, Eating Disorder Not Otherwise Specified (EDNOS-BN) and Other Specified Feeding and Eating Disorder (OSFED-BN).

**Results:**

Findings from 18 studies (seven randomised controlled trials, three secondary analyses, eight single-arm studies) encompassing 710 participants were synthesised. All studies were conducted in the USA (10/18, 55.6%), UK (4/18, 22.2%), and mainland Europe (4/18, 22.2%). Most were conducted in an outpatient setting (14/18, 77.8%), with the remainder conducted in a day hospital (2/18, 11.1%), mixed outpatient/day hospital (1/18, 5.6%), or residential (1/18, 5.6%) setting. Family-focused therapies (10/18, 55.6%) and cognitive behavioural therapies (10/18, 55.6%) were most represented. Both were associated with improvements in BN psychopathology, comorbid difficulties and parent/caregiver factors. Weak evidence in favour of adjunctive therapies and Fluoxetine were reported.

**Discussion:**

There is a striking paucity in adolescent bulimia nervosa intervention research. Whilst family-focused and cognitive behavioural therapies show promise, the evidence base is relatively small. Most studies had small sample sizes and were conducted with predominately White, female participants. Very little data are available regarding parent/caregiver outcomes. Future research focusing on theory-driven mechanisms that target the broader presentation of BN are needed.

**Supplementary Information:**

The online version contains supplementary material available at 10.1186/s40337-025-01236-8.

## Abstract (plain language summary)

This study aimed to systematically scope the available evidence for adolescent Bulimia Nervosa (BN) intervention in terms of symptom outcomes, related difficulties and parent/caregiver factors. Five main and three grey literature databases were searched on 4th September 2024. Relevant peer-reviewed journal articles, dissertations and book chapters were included. Findings from 18 studies (seven randomised controlled trials, three secondary-analyses, eight single-arm studies) encompassing 710 people were synthesised. All studies were conducted in the USA, UK or mainland Europe. Family-focused therapies and cognitive behavioural therapies were most represented. Both were associated with improvements in adolescent BN symptoms, comorbid difficulties and parent/caregiver factors. One study looked at the impact of medication (Fluoxetine 60mg/daily), with promising findings. There is a striking lack of adolescent bulimia nervosa intervention research. Whilst family-focused and cognitive behavioural therapies show promise, the evidence base is relatively small. Most studies had small sample sizes with participants who identified as White and female. Very little data are available on the impact of treatment on parents/caregivers. More higher quality studies, with larger and more diverse participants are needed.

**Supplementary Information:**

The online version contains supplementary material available at 10.1186/s40337-025-01236-8.

## Background

Bulimia Nervosa (BN) typically arises during adolescence or early adulthood [1]. It is estimated that up to 3% of females and more than 1% of males suffer from BN during their lifetime [2]. The age-standardized prevalence estimate of BN in children and adolescents in the recent Global Burden of Disease study 2019 was 58.55 (95% UI: 32.15–101.10) per 100,000 [3]. Population based studies using broader disordered eating criteria report BN rates as high as 14–22% [1, 4, 5].

BN has significant psychological and social sequalae, which may impact the developing adolescent and their family. A 2011 cross-sectional study of 10,123 adolescents with BN reported 88% to have at least one comorbid DSM-IV Axis-1 disorder, with 53% reporting lifetime suicidality and 35.1% reporting at least one historical suicide attempt [5]. Furthermore, 78% acknowledged BN to have contributed to some level of functional impairment. Left unaddressed, these comorbidities remain a significant issue for adults with BN, with evidence of high rates of affective disorders [6], alcohol and substance use disorders [7], attention deficit hyperactivity disorder (ADHD) [8] and personality disorders [9], with borderline personality disorder traits particularly common amongst adult women with BN [10]. This reinforces the importance of early adolescent intervention for BN and its associated comorbidities, particularly when considering comorbidity as a risk factor for increased mortality amongst adults with BN [11].

There is notable disparity regarding the treatment evidence base between adult and adolescent BN. Whilst numerous robust randomised controlled trials (RCTs) demonstrate the efficacy of treatments amongst adults [12], research into adolescent BN treatment efficacy remains relatively sparse [13, 14]. It is also unclear to what extent conclusions drawn from adult BN research can be applied to adolescents. A 2004 Cochrane review supported the efficacy of cognitive behavioural therapy (CBT), specifically CBT-BN in the treatment of adults with BN, informing current National Institute for Health and Care Excellence (NICE) guidelines [15]. Other psychological therapies such as interpersonal psychotherapy have also being reported as efficacious over the longer term [16]. Previous narrative reviews of adolescent BN treatment [14, 17] as well as a more recent systematic review of broader adolescent eating disorder treatments [13] further highlighted the scarcity of robust research, identifying only one open medication trial [18] and four RCT’s [19–22].

Whilst several reviews exist, most are now relatively old or overly stringent in their inclusion criteria (e.g. RCTs only) [13]. They also typically do not include the impact of treatment on co-morbid and family/caregiver factors. Given the dearth of adolescent BN RCTs, a more exhaustive, systematic scoping review of adolescent BN studies is needed. Specifically, a review of all currently available data, including lower quality and adjunctive intervention studies, which may previously have been excluded, is needed. The current review aimed to meet this need by systematically and comprehensively scoping the quantitative literature. The current review has four aims:

1. To review the impact of interventions on the psychological and behavioural symptoms of adolescent bulimia nervosa
2. To review the impact of interventions on comorbid psychiatric factors (i.e. mood, anxiety, etc)
3. To review the impact of interventions on family/parental/caregiver outcomes
4. Provide recommendations for future research

## Methodology

A systematic scoping review methodology [23] was used for this review, including the Preferred Reporting Items for Systematic Reviews and Meta-Analyses (PRISMA) [24]. This was deemed the most appropriate methodology to meet the study aims as it allowed for the inclusion of a range of study types, quality and interventions targeting BN symptoms (e.g. psychological, medication, physical, etc.). Systematic scoping review methodology allows for a broader coverage of the available literature and provides an overview of the available literature, rather than the more specific remit of a systematic review [25]. Study methodology was designed by both authors (ML, JB) using the PICOS (population, intervention, comparison, outcome, study design) framework [26]. ML implemented the initial search strategy and study selection, inconsistencies regarding study selection were cross referenced against initial inclusion criteria and resolved by consensus discussions. Zotero software was used during this process. ML completed data extraction.

### Eligibility criteria

Eligibility criteria are presented in Table 1.

**Table 1:** Scoping review eligibility criteria

	Included	Excluded
Publication type	Peer-reviewed articles Book chapters Dissertations	Published abstracts
Language	English	Non-english Language
Study Objectives	Investigates the impact of an intervention on adolescent bulimia nervosa outcomes, including: Explicit focus on eating disorder outcomes (e.g., formal outcome measures, frequency of disordered eating/compensatory behaviours) Explicit focus on comorbid symptoms Explicit focus on parent and family outcomes	
Methodology/Design	Any quantitative experimental design Any date Must include adequately described methodology appropriate to research question Must include quantitative measures of the relationship between intervention and eating disorder outcomes Must include a measure of eating disorder symptoms pre and post treatment	Qualitative study design Systematic or scoping reviews Meta-analyses Case report design Satisfaction or acceptability data only No data collection methodology or analysis reported Case series without reported means
Sample	Children and Adolescents (up to 25 years old) Formal diagnosis of either Bulimia Nervosa or eating disorder not otherwise specified (EDNOS) or Other Specified Feeding and Eating Disorder (OSFED)-BN type Any treatment setting	Sample aged under 25 but specifically exclude adolescent cohort i.e. young adults (18–25 year old’s) only Multi-diagnostic adolescent eating disorder samples where data are not reported separately per diagnosis Mixed adolescent and adult studies where data are not reported separately for adolescents

### Search strategy

Five main databases (Medline, PsychInfo, Embase, CENTRAL, CINAHL) and three grey literature databases (SCOPUS, Web of Science and ProQuest Dissertations and Theses Global) were searched using variations of the terms “adolescent”, “bulimia nervosa” and “intervention” on 4th September 2024 (see Supplementary Material 1 for exact search terms). Reference lists of identified studies were also screened for any additional relevant papers meeting the inclusion criteria.

### Selection process

**Fig. 1:**
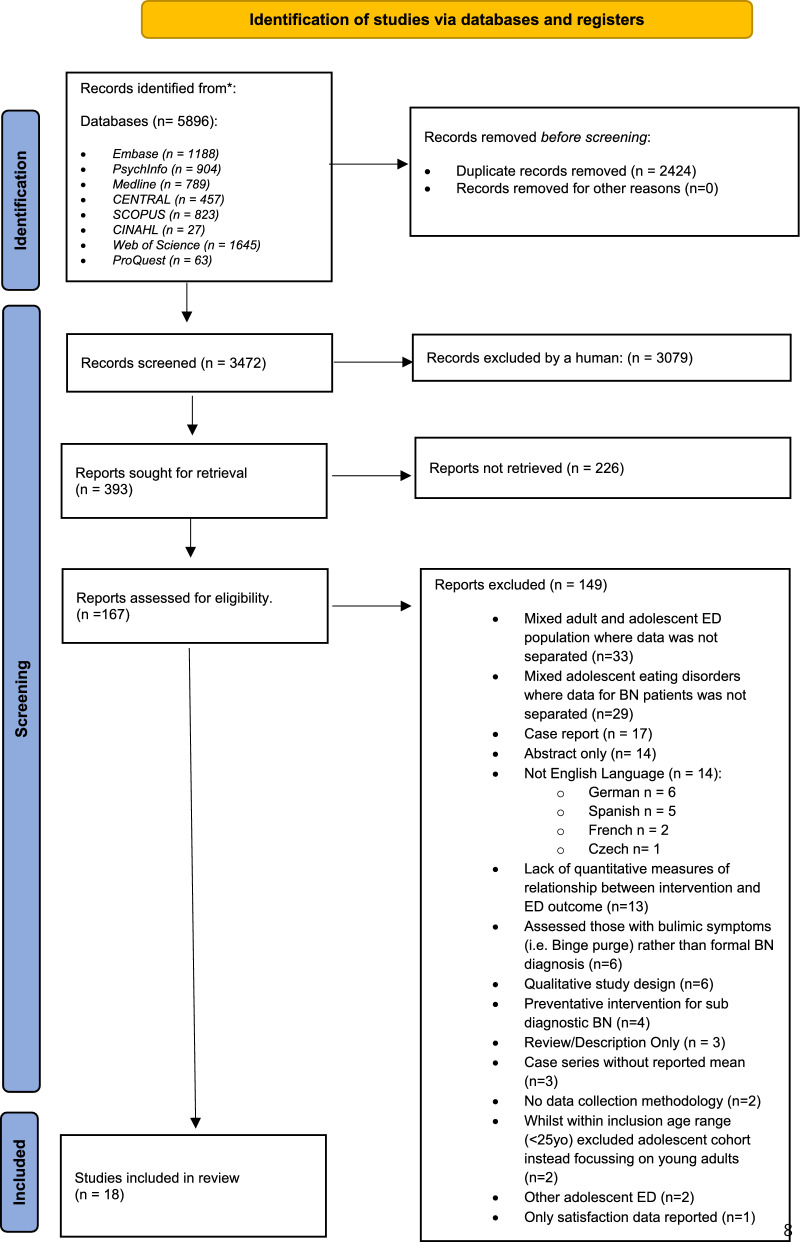
PRISMA flow diagram

ML conducted the initial search. Duplicates were then manually removed and remaining titles and abstracts reviewed by ML. Full-text citations and reference lists of the remining relevant manuscripts were screened by both authors for further eligibility before reaching agreement on the included manuscripts (Fig. 1 PRISMA). Consensus was reached via an iterative process during three, separate, one-hour meetings.

In keeping with other research, studies that were predominantly adolescent focused but included participants up to the age of 25 were included. However, exclusively ‘young adult’ studies recruiting individuals > 18 (despite all participants being under age 25) where excluded from this review. This resulted in the removal of four studies [27–30].

### Data extraction, charting, and categorisation

Included studies were charted by study aims. Tables were developed by ML in consultation with JB to determine variable extraction. For all three aims this included organising data by study design (RCT, RCT secondary analyses and single-arm studies), study setting (outpatient, day hospital, residential, inpatient), participant demographics and intervention characteristics, including intensity and duration. Aim 1 examined change in BN symptoms, aim 2 change in comorbid individual factors, and aim 3 change in parent/carer factors. All data were compared from baseline to end-of-treatment and follow-up if available.

### Risk of bias

Risk of bias was assessed using the Mixed Method Appraisal Tool [31]. While systematic scoping review guidance does not require risk of bias assessment [25, 32], it was completed for the current review as it was deemed useful in understanding the available data.

## Results

### Study selection and characteristics

5896 papers were initially identified from the search strategy. Subsequent removal of duplicates and manuscripts not meeting eligibility criteria resulted in 18 studies being included in the final review (Fig. 1 for PRISMA).

The total sample size of the included studies was 710 young people (mean age = 16.89 years, range 12–21 years, 96.7% female). Three studies were secondary analyses of an included RCT [20]. As these duplicated participant numbers and demographics, these data were excluded from overall sample calculations. See Tables 2 for a summary of study characteristics, demographics, design and follow-up timepoints.

**Table 2:** Summary of study characteristics and demographics

Author	Country	Year	Design	N	Age^b^	Range	%F	Intervention focus^c^			Setting	Tx Duration	Follow-up post-EOT
								Family-focused	Individual				
*Randomised controlled trials*													
Field et al	USA	1998	RCT	24	nr	16–21	100%	-	Massage + TAU	TAU	Residential	5 weeks	Nil
Johnson et al	USA	1998	RCT	13	17	nr	100%	EFFT	Group-CBT	–	OP	~ 10 weeks	Nil
Le Grange et al	USA	2007	RCT	80	16.1	12–19	98%	FBT-BN	SPT	–	OP	6 months	6 months
Le Grange et al	USA	2015	RCT	109	15.8	12–18	94%	FBT-BN	CBT-A	–	OP	6 months	6 and 12 months
Schmidt et al	UK	2007	RCT	85	17.65	13–20	97.6%	FT-BN	CBT-GSH	–	OP	~ 6 months	6 months
Stefini et al	Germany	2017	RCT	81	18.7	14–20	100%	–	CBT	PDT	OP	1 year	12 months
Wagner et al	Austria / Germany	2013	RCT	29	19.31	16–21	100%	–	CBT-GSH	CBT-BiB	OP	4–7 months	18 months post-baseline
*RCT secondary analysis*													
Matheson et al	USA	2024	RCT SA	51^a^	–	–	–	FBT-BN^a^	CBT-A^a^	–	OP	6 months	6 and 12 months
Reilly et al	USA	2022	RCT SA	109^a^	–	–	–	FBT-BN^a^	CBT-A^a^	–	OP	6 months	6 and 12 months
Valenzuela et al	USA	2018	RCT SA	109^a^	–	–	–	FBT-BN^a^	CBT-A^a^	–	OP	6 months	6 and 12 months
*Single-arm study*													
Dodge et al	UK	1995	Single-arm	8	16.5	14–17	100%	FT-BN	–		OP	1–17 months^d^	1-year post-baseline (ranged 11–4 months post-EOT)^d^
Kotler et al	USA	2003	Single-arm	13	16.2	12–18	100%	–	Medication	–	OP	8 weeks	Nil
Lazaro et al	Spain	2010	Single-arm	44	16.3	13–18	90.9%	–	Group-CBT/BT	–	Day Hospital	8 weeks	Nil
Lebow et al	USA	2022	Single-arm	8	16.1	15–18	75%	–	ICAT-A	–	OP	10–27 sessions	Nil
Martinez-Mallen et al	Spain	2007	Single-arm	25	16.7	14–19	100%	–	Cue exposure	–	Day Hospital / OP	6 weeks	6 months
Murray et al	USA	2015	Single-arm	40	15.7	14–17	100%	FBT + DBT	–	–	PHP	~ 77 days^e^	Nil
Pretorius et al	UK	2009	Single-arm	101	18.8	13–20	97%	–	Online-CBT	–	OP	8 sessions^f^	3 and 6 months post-baseline
Stewart et al	UK	2021	Single-arm	50	15.6	nr	98%	MFT-BN	–	–	OP	4 months	Nil
			Totals:	710	16.89	12–21	96.7%	7	12				

BiB, bibliotherapy; BT, behavioural therapy; CBT, cognitive behavioural therapy; CBT-A; adolescent adapted CBT; DBT, dialectical behaviour therapy; EFFT, emotionally focused family therapy; EOT, end of treatment; F, female; FBT-BN, Family-Based Treatment for Bulimia Nervosa; FT-BN, family therapy for bulimia nervosa; GSH, guided self-help; ICAT-A, integrative cognitive affective therapy for adolescents; IP, inpatient; MFT-BN, multi-family therapy for bulimia nervosa; nr, not reported; OP, outpatient; PDT, psychodynamic therapy; PHP, partial hospitalisation program; RCT, randomised controlled trial); SA, secondary analysis; SPT, supportive psychotherapy; TAU, treatment as usual; UK, United Kingdom; USA, United States of America

^a^Participant and treatment characteristics not included in totals due to study being a secondary analysis of an already reported on RCT [20]

^b^Mean reported

^c^Two interventions listed if study design included a comparison

^d^All but on participant received 1–10 months of intervention. The remaining one participant received 17 months of treatment. This participant is not included in follow-up duration

^e^6 day/week program with reducing number of days/week attended

^f^Weekly completion of sessions encouraged

A narrative synthesis of the 18 studies is reported below, with data from seven RCTs, three RCT secondary analyses, and eight single-arm studies. Three studies were classified as RCT’s based on methodology reporting “random allocation” to experimental or control group, despite lacking details around randomisation, blinding or group comparability [33–35]. Whilst a number of studies also included qualitative data, this was not commented on, as it was outside the aims of this review.

The majority of studies were from the USA (n = 10, 55.5%). Whilst two studies included a sample size greater than 100 (n = 2, 11.1%), the remaining studies were far smaller, with 38.8% (n = 7) having less than 40 participants and 22.2% (n = 4) less than 20. Most studies reported data from interventions delivered in an outpatient setting (n = 14, 77.7%), three were in a residential/day hospital setting (n = 3, 16.66%) and one in a mixed outpatient/day hospital setting (n = 1, 5.5%). Seven studies included adolescents with diagnosed BN (n = 7, 38.8%), six included a mixed full, subthreshold or partial BN sample (n = 6, 33.3%) and five a combined adolescent BN and Eating Disorder Not Otherwise Specified (EDNOS-BN) or Other Specified Feeding and Eating Disorder (OSFED-BN) samples (n = 5, 27.7%). One included a mixed adolescent ED sample (AN, BN, EDNOS) (n = 1, 5.5%) [36] and one a mixed adult and adolescent BN sample (n = 1, 5.5%) [35]. Both were included in this review as they reported on adolescent BN outcomes separately. Ten studies examined a family-focused intervention (n = 10, 55.5%). Regarding risk of bias, six studies were rated as high, eight as moderate and four as low in quality. See Supplementary Material 2 for details.

### Narrative synthesis

#### Physical and psychological symptom outcomes (aim 1)

See Table 3 for a summary of data related to psychological and behavioural symptom outcomes. Further details are presented in an extended table provided in the Supplementary Material.

**Table 3:**
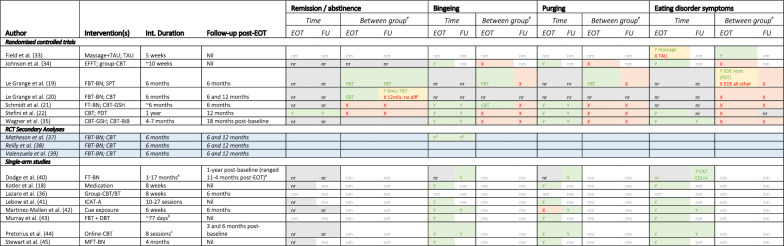
Significant differences in symptoms of bulimia nervosa across treatments and follow-up (aim 1)

Cell colour key: Green (Y), Significant difference detected; Red (X), no significant difference detected; Yellow, Mixed outcomes— some significant (Y), some not significant (X) differences detected; Grey, not reported; White, not measured; Blue, secondary analysis of included RCT [20]

^a^All but on participant received 1–10 months of intervention. The remaining one participant received 17 months of treatment. This participant is not included in follow-up duration

^b^6 day/week program with reducing number of days/week attended

^c^Weekly completion of sessions encouraged

^d^Loss of control eating data

^e^For studies that reported a significant difference between groups the superior treatment is named in the cell

6mfu, 6-month follow-up; 12mfu, 12-month follow-up; BiB, bibliotherapy; BT, behavioural therapy; CBT, cognitive behavioural therapy; DBT, dialectical behaviour therapy; EAT, Eating Attitudes Test; EDE, Eating Disorder Examination; EDI, Eating Disorder Inventory; EOT, end-of-treatment; FBT-BN, family-based treatment for bulimia nervosa; FT-BN, family therapy or bulimia nervosa; FU, follow-up; GSH, guided self-help; ICAT-A, integrative cognitive affective therapy for adolescents; nm, not measured; nr, not reported; PDT, psychodynamic therapy; RCT, randomised controlled trial; SPT, supportive psychotherapy

##### Randomised controlled trials (RCTs)

Seven RCTs with varying sample sizes and interventions were identified (see Table 3). Four larger (N = 80–109), well cited RCTs [19–22] and three smaller (N = 13–29), lower quality RCTs [33–35] were included. Of note, the latter three studies were categorised as RCTs in this review based on reference to “randomisation” of participants in their methodologies. However, none commented on the blinding or randomisation process. The majority of RCTs were conducted in an outpatient setting (n = 6, 85.7%). The remaining one was in a residential setting [33].

##### Family-focused interventions

Data from the four identified RCTs are generally supportive of family-focused interventions as effective in reducing BN symptoms. Data suggests FBT-BN and EFFT may lead to greater symptom reduction than CBT-based interventions or supportive psychotherapy, although differences in outcome are relatively modest. One RCT suggests guided self-help CBT may be slightly more efficacious at end-of-treatment, although findings are mixed, and differences disappear at follow-up.

The largest, two-site, RCT [20] randomised 109 adolescents with BN or partial BN (binge/purge episodes more than once per week for six months) to six months of either manualised outpatient Family Based Treatment (FBT-BN) [46] or an adapted version of adolescent CBT (CBT-A) [47]. Individual CBT-A focused on body image and weight related cognitions and behaviours, whilst FBT-BN centred on supporting parents to take charge and manage their young person’s disordered eating behaviours early in treatment and then supporting the family to transition to developmentally appropriate independence. Significantly more young people were abstinence from bingeing and purging in the FBT-BN group compared to CBT-A at end-of-treatment. Whilst FBT-BN remained superior, both groups showed sustained improvement at six-month follow-up, with no difference in abstinence rates between group at 12-month follow-up. Dropout rates of 10% (EOT), 38% (six months) and 36% (12 months) were a limitation.

Two further RCT’s [19, 21] also compared family therapies against individual psychotherapy. The larger of these [21] randomised 85 adolescents with BN or EDNOS-BN to six months Family Therapy (FT-BN) versus 10 sessions of guided self-help CBT (CBT-GSH). Primary outcome was objective abstinence from bingeing and vomiting at end-of-treatment. In contrast to the above study, results demonstrated that CBT-GSH was superior to FBT-BN, albeit only regarding bingeing abstinence at end-of-treatment. This difference was not sustained at 12 months (six-month follow-up). CBT-GSH also showed earlier improvement in bingeing (at six months) compared to FT-BN. Of note, no significant differences were observed between groups for purging or primary outcome of bingeing *and* purging at end-of-treatment or 12 months (six-month follow-up).

Le Grange et al.'s [19] earlier RCT compared FBT-BN against individual supportive psychotherapy (SPT), a non-specific supportive treatment adapted from an adult BN treatment. 80 adolescents with BN or partial BN were randomised to six months of either intervention. Primary outcome was the proportion of participants in remission or partial remission, defined as those meeting all DSM-IV criteria except the binge or purge frequency of once per week for six months, at end-of-treatment. Secondary outcome was binge/purge frequency and subscale scores on the Eating Disorder Examination (EDE) [48]. FBT-BN was associated with significantly higher rates of abstinence from bingeing and purging compared to SPT at end-of-treatment. This was sustained at six-month follow-up. Nevertheless, overall abstinence rates declined in both groups at six-month follow-up. Regarding secondary outcomes, random regression analysis showed main effects in favour of FBT-BN on all EDE subscales with a reduction in core bulimic symptoms observed more quickly in the FBT-BN group.

A smaller RCT (N = 13) explored emotionally focused family therapy (EFFT) [34]. Thirteen adolescents with BN were randomised to 10 sessions of EFFT (n = 9) or 10 weeks of group CBT (n = 4). Primary outcome was change in Diagnostic Survey for Eating Disorders (DSES) [49], Eating Disorder Inventory (EDI) [50] and Bulimic Symptom Checklist (BSCL) [51] scores. Both EFFT and group CBT were associated with a decrease in bulimic symptoms, measured using the EDI and BSCL scores, with no differences between groups. Of note, 23 people declined to participate due to not wanting their family members involved in treatment and/or that their families were unaware of their eating disorder.

##### Individual interventions

Data from RCTs examining outcomes of individual interventions suggest CBT, psychodynamic therapy and guided self-help CBT may be effective at supporting BN symptom reduction.

One larger RCT randomised participants (N = 81) with full or partial BN (defined as bingeing and purging less than two times per week over three months) to 12 months of two manualised individual interventions; psychodynamic therapy (PDT) and CBT [22]. Both interventions used a disorder specific, symptom orientated approach, albeit differed with regards their respective focus on emotions, behaviour, and cognitions. Primary outcome was remission, defined as a lack of DSM-IV BN/partial BN diagnosis at end-of-treatment. Secondary outcomes were frequency of binge/purge episodes EDE and Eating Disorder Examination Questionnaire (EDE-Q) German version [52] subscales and global scores at end-of-treatment and 12-month follow-up (24 months). No between group difference in remission rates was reported at end-of-treatment. Significant improvements in secondary outcome measures were observed in both treatments, and improvements were maintained between end-of-treatment (12 months) and 12-month follow-up (24 months). Small between group effect sizes were observed for binge/purge behaviours in favour of CBT and for eating concern in favour of PDT. Of note, this study was not powered to be an equivalence trial, potentially accounting for lack of between group differences. A dropout rate of 30% was also reported, which the authors attributed to the relatively long intervention length. Dropout was higher in the CBT group (38.5%).

A smaller study explored the efficacy of CBT guided self-help [35]. Adolescent outcomes were compared to adults with BN, with the authors hypothesising treatment to be equally efficacious. The mixed adult/adolescent sample consisted of 29 adolescents and 97 adults with BN or EDNOS-BN. Only adolescent (age range 16–21) outcomes are reported in the current review. Participants were randomised to two forms of guided self-help: internet-based CBT guided self-help (INT-GSH) or bibliography-based CBT guided self-help (BIB-GSH) for four to seven months. Main outcome measures were remission rates and eating disorder psychopathology measured using the Questionnaire Anamnesetique pour les Troubles Alimentaire (QATA) [53] and the Eating Disorder inventory-2 (EDI-2) [54]. Results for both groups were combined due to lack of between group differences, with 44% of adolescents in remission or abstinent by end-of-treatment (seven months) and 55% in remission or abstinent at follow-up (18 months). Over time there was a significant improvement in mean monthly binge eating, vomiting and fasting, with the highest decrease observed during the first four months of treatment. On the EDI-2, all sub-scale scores except perfectionism improved over time, with highest decrease within the first four months of therapy, and then evening out or slightly increases at the end of follow-up. On the EDI-2, no differences between the adolescent and adult groups were reported, except on maturation fears for which the adolescent group scored significantly higher. As such, adolescent only EDI-2 score changes are not reported. Analysis of the whole sample (combined adolescent and adult data) demonstrated an improvement on all EDI-2 subscales, except perfectionism, over time. The greatest improvement occurred within the first four months of treatment. Whilst supporting the use of technology assisted CBT in improving BN psychopathology, the lack of separate INT-GSH and BIB-GSH outcome reporting, absence of specific EDI-2 adolescent BN statistical analysis, small sample size and treatment attrition (39.8% of cohort did not report outcome, although it unspecified what proportion of these were adolescents) makes interpretation of these data limited.

##### Residential treatment

The only residential RCT [33] evaluated adjunctive massage therapy versus treatment as usual, which included 30–40 group therapy sessions per week. 24 female with BN were randomly assigned to received 10 massages over five weeks. They completed the EDI [50] on the first and last day of the five-week intervention. The massage group had significantly reduced scores on the EDI total score and several subscales (drive for thinness, bulimia, dissatisfaction, ineffectiveness, perfectionism, interpersonal distrust, interoceptive awareness, and maturity fears) compared to the treatment as usual group. The authors hypothesised that massage therapy may raise patients’ awareness of their bodies, helping them to challenge body image dissonance. Whilst small, with limited reporting of outcome data, findings reinforce the importance of approaching adolescent BN treatment holistically.

##### Single-arm studies

Six case series [18, 40, 42–44, 55], one secondary analysis [37] and one retrospective audit [45] were included. see Table 4 for details. Most (n = 6/8, 75.0%) were conducted in an outpatient setting, with one in a partial hospitalisation program [43] and another in a mixed inpatient/outpatient setting [42]. Five studies explored outcomes of family-focused interventions.

**Table 4:** Impact of interventions on comorbid individual and parent/carer factors (aim 2 and 3)

Author (year) [Country], Design (Setting)^a^	Mean age (SD), Sample n, % female, race/ethnicity, SES, Diagnosis	Intervention	Young person domain (measure)	Young person outcome	Parent domain (measure)	Parent outcome
*Randomised controlled trials*						
Field et al. (1998) [USA] RCT (Residential)	nr (nr, range 16–21) N = 24 F: 100% E: Hispanic 68%, Non-Hispanic White 32%, SES: Middle to upper SES [2.2 on Hollingshead Index]) DSM-III-R BN (100%)	Massage therapy + TAU vs TAU 10 massages/5 weeks	Depression (POMS-D, CES-D) Anxiety (state; self-report) (STAI) Anxiety (observational) (BOS)	Pre-post massage on first day Significant decrease on depression (POMS-D; *p* = .001, ES nr), self-report state anxiety (STAI-state; *p* = .001, ES nr) and observe anxiety (BOS; *p* = .005, ES nr). No significant effects for the control group Pre-post massage on last day Significant decrease on depression (POMS-D; *p* = .001, ES nr), self-report state anxiety (STAI-state; *p* = .001, ES nr) but not observed anxiety (BOS; p nr, ES nr). No significant effects for the control group Pre-post 5-week (10 massages) intervention plus TAU Significant decrease on depression (CES-D) in both massage (*p* = .001, ES nr) and control groups (*p* = .05, ES nr)	Nil measured	Nil measured
Johnson et al. (1998) [USA] RCT (OP)	17 (nr) N = 13 F: 100% E: nr SES: nr) DSM-III-R BN (100%)	EFFT vs group CBT 10 weeks CBT Group Therapy vs 10 sessions of EFFT	Depression (BDI) General Psychiatric Symptoms (SCL-90-R)	Both treatment arms Significant decrease in general psychiatric symptomatology after both CBT and EFFT on SCL-90-R and BDI (*p*’s < .05, EF nr). SCL-90-R subscales not reported. No between group effect EFFT group only: Reduction on BDI (*p* < .05, EF nr) and SCL-90-R obsessive compulsivity (*p* < .05, EF nr), interpersonal sensitivity (*p* < .01, EF nr), depression (*p* < .05, EF nr), hostility (*p* < .05) and psychoticism (*p* < .05, EF nr) subscales	Nil measured	Nil measured
Le Grange et al. (2007) [USA] RCT (OP)	16.1 (1.7) N = 80 F: 98% E: White 64%, Hispanic 20%, African American 11%, Other 5% SES: nr DSM-IV BN (46.2%) Subthreshold BN (53.8%)	FBT-BN (n = 41) vs SPT (n = 39) 20 sessions/6 months	Depression (BDI) Self-esteem (RSES)	Mixed effects linear regression models showed no differences between groups at EOT or 6mfu in self-esteem (RSES; *p* = 0.33, ES nr) and depression (BDI; *p* = 0.72, ES nr)	Nil measured	Nil measured
Le Grange et al. (2015) [USA] RCT (OP)	15.8 (1.5) N = 109 F: 94% E: 46% “ethnic minority” SES: Income > 100 K (CBT-A 40%, FBT-BN 37%), Parent with college degree: [CBT-A 74%, FBT-BN 65%]) DSM -IV BN/Partial BN (100%)	FBT-BN (n = 51) vs CBT (n = 58) vs SPT (n = 20; not included in analysis) 18 sessions/6 months	Depression (BDI) General obsessive–compulsive symptoms (CYBOCS) ED-specific obsessive–compulsive symptoms (YBC-ED) Self-esteem (RSES)	Using mixed effects modelling, depression (BDI) was the only domain that showed a significant group difference (*p* = .049, d = 0.361) at EOT. The FBT-BN group had lower BDI scores compared to the CBT-A group. No significant between group effects observed for obsessive–compulsive symptoms (CYBOCS) or ED specific obsessive–compulsive symptoms (YBC-ED) at any timepoint (EOT, 6mfu, 12mfu) NB: Self-esteem (Valenzuela, et al. 2018) and obsessive–compulsive (Reilly et al. 2022) data from this trial are presented in secondary analyses	Family Functioning (FES)	Only baseline data reported
Stefini et al. (2017) [Germany] RCT (OP)	18.7 (1.9) N = 81 F: 100% E: nr SES: nr DSM-IV BN (77.7%), DSM-IV Partial BN (22.3%)	CBT (n = 39) vs PDT (n = 42) Up to 60 sessions/1 yr	General Psychiatric Symptoms (SCL-90-R)	Significant reduction in general psychiatric symptoms (SCL-90-R) in both groups—CBT group (within subjects *p* < .001, d = 0.51); PDT group (within subjects *p* < .001, d = 0.24). No difference between groups (*p* = .35, d = 0.16)	Nil measured	Nil measured
*RCT secondary analysis*						
Reilly et al. (2022) [USA] Secondary Analysis (Le Grange RCT 2015) (OP)	As per Le Grange, et al. (2015)	As per Le Grange, et al. (2015)	General Obsessive–Compulsive symptoms (CYBOCS) ED Specific Obsessive–Compulsive symptoms (YBC-ED)	Multilevel models showed no change in general obsessive–compulsive symptoms (CYBOCS) over time in either treatment (*p* = .155, R^2^ = .01) Significant decrease in ED specific obsessive–compulsive symptoms (YBC-ED) throughout treatment and follow-up, irrespective of treatment type (*p* < .001, R^2^ = .03)	Nil measured	Nil measured
Valenzuela et al. (2018) [USA] Secondary Analysis (Le Grange RCT 2015) (OP)	As per Le Grange, et al. (2015)	As per Le Grange, et al. (2015)	Depression (BDI) Self-esteem (RSES)	Significant reduction in depression (BDI) in both groups (*p* < .001, estimate = − 3.18 [95%CI − 3.78, − 2.57]). No difference between groups (*p* nr, estimate = − 0.11 [95%CI − 0.72, 0.49]) Significant improvement in self-esteem (RSES) in both groups (*p* < .001, estimate = − 3.30 [95%CI 2.35, 4.25]). No difference between groups (*p* nr, estimate = 0.25 [95%CI − 0.70, 1.20])	Nil measured	Nil measured
*Single-arm studies*						
Kotler et al. (2003) [USA] Case Series (OP)	16.2 (1.0) N = 10 F: 100% E: Caucasian 50%, Hispanic 30%, Asian American 10%, Indian 10%, SES: nr DSM-IV BN (80%) EDNOS (20%)	Fluoxetine 60 mg/day with supportive psychotherapy 8 weeks	Depression (BDI) Anxiety (SCARED)	Significant decrease on anxiety (SCARED; *p* < .05, ES nr), but not depression (BDI; p nr, ES nr)	Nil measured	Nil measured
Lazaro et al. (2010) [Spain] Case Series (Day Hospital)	16.3 (1.1) N (BN group): 44 F: 90.9% E: nr SES: nr Mixed ED sample (AN/BN/EDNOS) DSM-IV BN (63.6%) EDNOS-BN (36.3%)	Structured behavioural self-esteem and social skills group therapy in day treatment programme 8 sessions/2 months	Self-esteem/concept (PHC-SCS) Self-esteem in eating disorders (SEED) Social Skills (BAS-3)	Significant improvement on 3 self-esteem/concept (PHC-SCS) subscales; intellectual/school status (*p* = .010, ES nr), physical appearance (*p* = .008, ES nr), freedom from anxiety (*p* = .040, ES nr). No change in behavioural adjustment (*p* = .582, ES nr), popularity (*p* = .141, ES nr) or happiness/satisfaction (*p* = .438, ES nr) subscales No change in self-concept (SEED) related others (*p* = .079, ES nr) or weight/shape (*p* = .054, ES nr) Significant improvements on 2 social skills (BAS-3) subscales; consideration for others (*p* = .015, ES nr), and social withdrawal (*p* = .042, ES nr). No change in self- control in social relations (*p* = .138, ES nr), social anxiety/shyness (*p* = .230, ES nr), or leadership (*p* < .001, ES nr). No change on consideration for others (*p* = .066, ES nr) subscales	Nil measured	Nil measured
Martinez-Mallen et al. (2007) [Spain] Case Series (OP/Day Hospital)	16.7 (SD 1.5) N = 25 F: 100% E: nr SES: nr DSM-IV BN (100%) (not responded to standard care)	Cue Exposure Program 12 session/6-weeks	Depression (BDI) Anxiety (state and trait) (STAI)	Significant reduction in state (STAI-state; *p* < .05, ES nr) and trait (STAI-trait; *p* < .05, ES nr) anxiety Significant reduction in depression (BDI; *p* < .05, ES nr) NB: p values are from one-way ANOVA using 3 timepoints (baseline, EOT, 6 m FU)	Nil measured	Nil measured
Murray et al. (2015) [USA] Case Series (PHP)	15.7 (1.11) N = 35 F: 100%, E: Caucasian 63.8%, Hispanic 14.5%, Asian 2.9%, Black 2.9%, Other 15.9% SES: nr BN (100%)	Integrated FBT and DBT 3-10 h/day, up to 6 days/week. Mean treatment length 77.18 days (SD 38.91)	Emotion regulation (DERS)	Significant improvement on 1 DERS subscale; access to emotion regulation strategies (*p* = .045, ES nr) at end-of-treatment. No significant change in global score or non-acceptance, goal difficulties, impulse difficulties, emotional awareness, lack of emotional clarity and global subscales of DERS (all *p*’s > .05, ES nr)	Parent eating-disorder-related self-efficacy (PVA)	Significant increase in eating-disorder-related parental self-efficacy (PVA) across treatment (*p* = .001, ES nr)
Stewart et al. (2021) [UK] Retrospective audit (OP)	15.6 (1.4) N = 50 F: 98% E: nr SES: nr BN (100%)	MFT-BN Weekly/4 months	Depression (RCADS-D) Anxiety (RCADS-A) Emotion regulation (DERS)	Significant reduction in depression (RCADS-depression; *p* = .011, d = − 0.78), anxiety (RCADS-anxiety; *p* = .025, d = − 0.64) and global emotion regulation (DERS-total; *p* = .001, d = − 2.17)	Parent depression (HADS) Parent anxiety (HADS) Parent negative experiences of caregiving (ECI)	Significant reduction in parental depression (HADS-depression; *p* = .045, d − 0.33) and negative experiences of caregiving (ECI; *p* = .026, d − 0.46). No change in parental anxiety (HADS-anxiety; *p* = .120, d = − 0.31)

6mfu, 6-month follow-up; 12mfu, 12-month follow-up; ANOVA, analysis of variance; BAS-3, Bateria de Socializacion; BDI, Beck Depression Inventory; BOS, Behaviour Observation Scale; CBT-A, CBT-A, adolescent adapted CBT; CES-D, Centre for Epidemiologic Studies Depression Scale; CYBOCS, Children's Yale-Brown Obsessive Compulsive Scale; DERS, Difficulties in Emotion Regulation Scale; ECI-N, Experience of Caregiving Inventory-negative subscale; EFFT, emotionally focused family therapy; ES, effect size; FBT-BN, Family-Based Treatment for Bulimia Nervosa; FES, Family Environment Scale; HADS, Hospital Anxiety and Depression Scale; PHC-SCS, Piers-Harris Children’s Self Concept Scale; POMS, Profile of Mood States; PvA, Parent versus Anorexia scale; RCADS, Revised Child Anxiety and Depression Scale; RSES, Rosenberg Self Esteem Scale; SCARED, Self-Report For Childhood Anxiety Related Disorders; SCL-90-R, Hopkin's Symptom Checklist-Revised; SEED, Self Esteem in Eating Disorders Questionnaire; STAI, State Trait Anxiety Inventory; TAU, treatment as usual; UK, United Kingdom; US, United States of America; YBC-ED, Yale-Brown-Cornell Eating Disorders Scale

^a^Six of the total number of studies identified in this review are not presented in this table as they did not report on any co-morbid individual or parent/career factors; two RCTs [21, 35], one RCT secondary analysis [37] and three single-arm studies [40, 44, 55]. Of note, Schmidt et al. [21] described collecting general psychopathology, family relationship and parental outcome (mental health and burden of caring) data in their methodology and an intention to report it separately, however, these data were not identified by the search strategy. In their methodology, Dodge et al. [40] describe collecting self-esteem (RSES) data but do report any findings. Lastly, Lebow et al. [41] collected baseline data on Depression (BDI-2), Anxiety (SCAS-C), Emotion regulation (DERS), and Self-esteem (RSES), but due to incomplete EOT data, change across treatment not reported

##### Family-focused interventions

Four studies investigated the impact of family-focussed interventions on BN symptoms. Three were conducted in an outpatient context, the other in a partial hospitalisation program. Data suggest family-focused interventions are associated with significant reductions in bingeing and purging behaviours as well as eating disorder psychopathology.

In an early exploratory study, Dodge et al. [40] examined the effect of family therapy for eight adolescents with BN. Treatment duration ranged from 1 to 16 sessions over 1–17 months. At the end of treatment, a significant reduction in bingeing, purging and laxative use was reported. On the Morgan Russell Outcome Scale adapted for BN [56] one adolescent achieved a good outcome (no binge/purge symptoms with body weight above 85% median Body Mass Index [%mBMI]), five had an intermediate outcome (binge/purge symptoms less than once per week with body weight above 85%mBMI) and two a poor outcome (binge/purge symptoms more than once a week and/or body weight less than 85%mBMI). A significant reduction was also reported on the Eating Attitudes Test (EAT 40) [57], as well as a reduction (significance not reported) on the EDI [50].

Matheson et al. [37] conducted a secondary analysis of an aforementioned RCT [20]. They explored loss of control eating outcomes for the subgroup of adolescents who received FBT-BN (N = 51). Episodes of loss of control eating reduced significantly during treatment with large effect size. Approximately half (49%) of the participants reported abstinence from loss of control eating at the end of treatment (six months). At six-month follow-up (12 months) a slight increase in loss of control eating was observed, with 41% of the total sample reporting abstinence. Of note, a large amount of missing data at follow-up assessment time points (> 40%) were reported. While 73% (n = 22/30) of those who completed 12-month follow-up (18 months) assessments reported abstinence from loss of control eating in the month prior, this finding is interpreted with caution due to data missingness.

In a retrospective chart review (N = 50), Stewart et al. [45] examined the impact of multi-family therapy for bulimia nervosa (MFT-BN). MFT-BN extends the Maudsley single-family therapy model (FT-BN) by offering treatment in a group-based context (cf [58, 59] for reviews). MFT-BN is offered over four months, comprising of weekly two-hour sessions. The authors reported a significant reductions in bingeing and purging behaviours (but not laxative misuse), as well as self-reported eating disorder symptoms measured using the EDE-Q [60]. When compared to adolescents with BN who received single-family therapy in the same clinic, dropout rates were lower in the MFT-BN group.

Murray et al. [43] conducted an open trial (N = 35) in their partial hospitalisation program based on the principles of family-based therapy (FBT) and Dialectical Behaviour Therapy (DBT) (cf [61] for description of program). The treatment program included individual, family, multi-family and parent-only sessions. It operated six days per week for 3–10 hours per day. At the end of treatment, significant reductions were observed on several EDE-Q subscales, including shape concerns, weight concerns and global score. There were also significant reductions in bingeing, purging and secret eating. No follow-up data were reported.

##### Individual interventions

Three single-arm studies examined outcomes of individual interventions. Data suggests individual treatment is associated with a reduction in bingeing, purging and psychological symptoms associated with BN.

Pretorius et al. [44] explored the efficacy of an internet-based CBT intervention. 101 young people (93 females, 3 males, age range 13–20) with BN (n = 61) or EDNOS with bulimic features (n = 40) were offered eight sessions of internet-based CBT consisting of three components (overcoming bulimia online programme, electronic message boards and email support). Participants were recruited through outpatient eating disorder clinics and via advertisement through an eating disorder charity. Significant improvements were observed in objective bingeing, vomiting and global EDE scores from baseline to three months, which was maintained at six months. A significant reduction in laxative misuse from baseline to six months was also reported. Whilst promising, most participants remained symptomatic at three- and six-month timepoints. The proportion of participants who were either abstinent or in the subclinical range for bingeing, purging or laxatives misuse was 9% at baseline, 25% at 3 months and 29% at six months. Of note, a substantial number of participants did not complete follow-up interviews (three months: 51.5% completion, six months: 62.3% completion).

A more recent, small (N = 8), feasibility study [55] examined the impact of integrative cognitive affective therapy for adolescents (ICAT-A) with BN or subthreshold BN (met criteria for Other Specified Feeding or Eating Disorder [OSFED-BN]). Six participants (75%) completed treatment (defined as progressing through all phases of treatment). Mean number of individual sessions was 16.2 (sd = 6.2, range = 0–27) and conjoint sessions was 5.2 (sd = 3.4, range 1–8). One family had a parent-only session. All completers reached full remission measured on the EDE. From baseline to end-of-treatment, large effect size reductions were reported for global EDE scores, compensatory behaviours and objective binge episodes with a moderate effect size reduction also reported for subjective binge episodes. Of note, due to the COVID-19 pandemic, treatment needed to transition to telehealth or hybrid in person/telehealth sessions for four out of the six completers. This may have led to significant variability in intervention experience. This shift to telehealth delivery also impacted completion of end-of-treatment self-report measures, with only two of the six participants completing these.

Lastly, one mixed outpatient/day hospital study explored cue exposure in the treatment of refractory adolescent BN [42]. This study included 25 adolescents with refractory BN (defined as non-response to 6–8 months of CBT and 60mg Fluoxetine per day). The aim of cue exposure was to diminish the conditioned response (craving) to the existence of a conditioned stimulus (food). Twelve sessions of cue exposure were offered over six weeks. At the end of treatment, a significant reduction was observed in binge eating episodes, as well as scores on the EDI-2 bulimic factor, Bulimia Test Revised (BULIT) [62] and EAT-26 [63]. These improvements were all maintained at follow-up. Whilst purging behaviours reduced, the difference between baseline and end-of-treatment was not significant. However, a significant reduction was observed between baseline and six-month follow-up**.**

##### Pharmacological interventions

One small (N = 10) outpatient psychopharmacology study was identified by the search strategy with promising findings. Adolescents (age range = 12–18) received eight weeks of Fluoxetine (60mg/day) alongside psychotherapy over eight weeks [18]. They had all received supportive psychosocial treatment prior to entering the study. Adolescents whose symptoms had not significantly improved (defined as a reduction in binge and purge frequency of greater than 50%) during this period then entered the Fluoxetine study. Results showed a significant reduction at end-of-treatment in weekly binge and purge episodes. All young people showed some improvement on the Clinical Global Impression (CGI) scale [64] (20% much improved, 50% improved and 30% slightly improved). Improved scores were also reported on the EDI (Bulimia Scale) and EAT 40, but not on the Body Shape Questionnaire [65]. Fluoxetine was generally well tolerated. The small participant number and short study duration limit interpretation of the data, particularly with regards to outcomes beyond the eight-week intervention period.

### Interventions impact on comorbid individual factors (aim 2)

Ten studies assessed the impact of treatment on broader comorbid psychiatric symptoms. See Table 4 for a summary of findings. Studies were heterogenous regarding intervention type, outcome measures, setting and design. Three RCTs [22, 33, 34], two RCT secondary analyses [38, 39] and five case series [18, 36, 42, 43, 45] were identified by the search strategy. Four were conducted in an outpatient setting, one on an inpatient unit, two in a day-hospital setting, and one had a mixed outpatient and day-hospital sample. See Table 4 for details.

#### Randomised controlled trials (RCTs)

Data from three larger [19, 20, 22] and two smaller [33, 34] RCTs suggests FBT-BN, CBT, psychodynamic therapy and EFFT are associated with improvements in broader psychiatric symptomatology. Adjunctive massage therapy in a residential program is also associated with improved anxiety and depressive symptoms.

In their trial comparing CBT and psychodynamic therapy, Stefini et al. [22] found a significant reduction with medium effect size for both groups in general psychiatric symptomatology measured using The Symptom Checklist-Revised (SCL-90-R) [66]. No between group differences were reported. Similarly, in the small trial comparing EFFT and CBT educational groups (N = 13), Johnson et al. [34] also examined changes in general psychiatric symptomatology using the (SCL-90-R), as well as depressive symptoms using the Beck Depression Inventory (BDI) [67]. In addition to changes in BN symptoms (see above), significant improvements were observed on both the SCL-90-R and BDI in both groups. Whilst separate group outcome data were not reported, authors advised no differential treatment effects were observed. Some specific outcomes were reported for EFFT including a reduction on all SCL-90-R subtests and decreased symptom severity on the BDI.

Le Grange et al. [19, 20] examined several co-morbid factors in their two RCTs. In the earlier trial comparing FBT-BN to supporting psychotherapy [19], no group differences in self-esteem (measured using the RSES) or depression (measured using the BDI) were observed at end-of-treatment (six-months) or six-month follow-up (12-months). In their more recent trial comparing FBT-BN to CBT-A [20], they found that depression (measured using the BDI-II) was the only domain that showed a significant group difference of medium effect size at end-of-treatment. The FBT-BN group had lower BDI scores compared to the CBT-A group. No significant between group effects were observed for general (CYBOCS) or ED specific obsessive–compulsive symptoms (YBC-ED) at any timepoint (end-of-treatment, six-month and 12-month follow-up). Time effects were not reported in the original paper and have been published in a recent secondary analysis described below [38]. They also collected self-esteem data in the RCT. Again outcomes were not reported in the original paper, rather they were published in another secondary analysis also described below [39].

In Field et al. [33] residential-based RCT of treatment as usual with or without five weeks of adjunctive massage therapy, changes in symptoms of depression and anxiety were reported in addition to eating disorder symptomatology. They assessed change pre (30m before)-post (30m after) individual massages on the first and last day of the five-week trial, as well as overall change from baseline to end-of-treatment (10 massages provided over a five-week period). Significant short-term (30m post-massage) reductions were observed in symptoms of depression measured using the Profile of Mood States subscale (POMS) [68] and state anxiety measured using the subscale of the State Trait Anxiety Inventory (STAI) [69] for both the first and last massage received. No significant short-term changes were reported in clinician-rated observed anxiety measured using the Behaviour Observation Scale (BOS) [70] or salivary cortisol. Between baseline and end-of-treatment (10 massages over five weeks), significant reductions were observed in symptoms of depression measured using the Centre for Epidemiologic Studies Depression Scale (CES-D) [71], dopamine and urinary cortisol, but not for norepinephrine or epinephrine.

### Impact of intervention on parent/caregiver factors (aim 3)

Two single-arm studies explored the impact of adolescent BN interventions upon caregiver factors [43, 45]. Two others collected baseline data but did not report changes across treatment [20, 21, 41]. See Table 4 for details.

From the available data, BN interventions may lead to improvements in parental self-efficacy, depressive symptoms and experiences of caregiving. Murray et al. [43] found statistically significant improvements in parental eating-disorder-related self-efficacy on the Parent versus Anorexia scale (PVA) [82] following an integrated FBT and DBT partial hospitalisation program. In Stewart et al.'s [45] MFT-BN retrospective chart review they reported a significant reduction in parental depression but not anxiety symptoms (measured using the Hospital Anxiety and Depression Scale [HADS] [83]).They also reported a significant reduction in the negative experiences of caregiving (measured using the Experience of Caregiving Inventory [ECI] [84]).

## Discussion

This systematic scoping review examined existing evidence for adolescent BN interventions. Despite the relatively broad research question and inclusion criteria, our findings echo that of previous narrative reviews [14, 17]. Namely, that there is a general paucity of high-quality clinical studies and robust evidence. This is in stark contrast to the adult BN literature in which a number of RCT’s have demonstrated moderate to strong evidence in support of both pharmacological and behavioural interventions [85], with CBT-BN consistently shown to be efficacious for this group [86, 87]. Relative to adolescent AN, BN has also attracted significantly less research, despite the reported threefold higher BN lifetime prevalence of 0.3% compared to 0.9%, respectively [5].

Regarding the primary aim, more than half the studies explored the efficacy of family-focused therapies (FBT-BN, FT-BN, EFFT, MFT-BN, integrated FBT/DBT informed partial hospitalisation program) with the remaining interventions being individual psychological therapies (predominantly CBT-focussed), pharmacological or adjunctive interventions (cue exposure, adjunctive massage therapy). The predominance of family-focused interventions is unsurprising considering the evidence base for adolescent AN [88] and recommendation in international guidelines [89].

Expectedly, findings from the current review demonstrate the strongest evidence is for family-focused interventions for adolescent BN. In single- and multi-family outpatient formats they are associated with improvements in BN symptomatology, as well as related co-morbid factors such as symptoms of depression, anxiety and emotion regulation. The strongest evidence comes from two of the larger RCT’s, both of which compared individual psychotherapy to FBT-BN. Both demonstrated FBT-BN to be associated with greater symptom reduction compared to CBT-A [20] and supportive psychotherapy [19]. These improvements were subsequently sustained, to variable degrees, at follow-up. Nevertheless, individual approaches were slightly improved at end-of treatment (but not follow-up) in one family-focused intervention study [21].

Despite findings being generally in favour of family-focused interventions, abstinence rates remained relatively modest at end-of-treatment, with some studies reporting a further deterioration during the follow-up period. This is important given abstinence is a predictor of longer-term recovery [90] and suggests there is still a way to go regarding treatment development. Unfortunately, this is not unique to the adolescent group. Abstinence rates at end-of-treatment in most studies ranging from 20 to 40%, which is similar to those reported in the adult literature [91]. Whilst this review did not explore acceptability data, some studies referenced recruitment difficulties due to participant hesitancy in involving family members in both discussions around diagnosis and treatment [21, 34]. Schmidt et al. [21] reported that 28% of participants refused participation due to not wanting to involve their families. Additionally, of the 23 individuals who declined to take part in Johnson et al. [34] EFFT study, 40% stated this was due to not wanting family involved in their treatment and 32% stated their family were not aware of their eating disorder. Conversely, Stewart et al. [45] noted lower dropout rates in their multi-family compared to single-family interventions in their retrospective chart review. This fits with qualitative data that the inclusion of family members in a group-based format is experienced as valuable [92]. More data are needed to better understand the experience of family-focused interventions for BN, barriers to engagement, as well as efficacy and acceptability of offering treatment in a multi-family format.

The current review also provides preliminary evidence for the use of individual, online, group-based treatments, psychopharmacotherapy, cue exposure, and adjunctive massage therapy (in a residential treatment setting) to support young people with BN. The most robust data supporting individual approaches comes from Stefini et al. RCT [22] comparing CBT and PDT. They observed a statistically significant decrease in ED pathology in both treatments. End-of-treatment remission rates (CBT 33.3% vs PDT 30.2%) were slightly lower than some family-focused studies and are similar to those seen in adult studies [91]. They also reported that improvements were maintained at 12-month follow-up (24 months post-baseline) [22]. Fluoxetine (60mg/daily) alongside brief psychotherapy (eight weeks) also showed promise in the only identified medication trial [18].

Whilst not specifically explored within this review, cost effectiveness was also cited as an added benefit of a guided self-help CBT interventions when compared to a family-focused intervention [21]. Positive outcomes with internet-based guided self-help CBT interventions were also reported in a number of smaller single-arm studies [35, 44]. Web-based interventions are particularly important when considering access to specialist ED treatments outside of larger cities and specialist treatment centres. The evidence for CBT as an alternative intervention for this group is also supported by a lack of difference between family-focused interventions and CBT at longer (12 month) follow-up [20].

Regarding broader comorbid and related difficulties, findings from the current review suggest family-focused interventions, individual interventions, group-based interventions, Fluoxetine and massage therapy are all associated with improvements in a range of factors; namely, anxiety, low mood, eating-disorder-related obsessive compulsive symptoms and self-esteem. Nevertheless, these data are mixed and several studies supporting these findings were conducted in higher levels of care (e.g. day programs or residential treatments). In higher levels of care, young people typically access a large number of interventions concurrently making it difficult to ascertain the specific input of each treatment component [93].

One interventions, an integrated FBT/DBT partial hospitalisation program [43], specifically aimed to address emotional dysregulation and BN psychopathology concurrently. Whilst there was no notable improvement in global DERS scores, the authors hypothesised BN symptoms, may in themselves, be a means of emotion regulation, meaning improvements in BN symptoms may potentially result in elevated emotional dysregulation. More data are needed to better understand the interplay between BN symptom presentation and emotion regulation.

Regarding changes in parent/caregiver factors, only two single-arm studies reported relevant data. Both offered family-focused interventions; one in a partial hospitalisation program [43] and one in a multi-family format [45]. Whilst small and uncontrolled, both noted improvements in family functioning, carer mental wellbeing and their experiences of caregiving. These findings are particularly important when considering the known mental health burden upon carers of adolescents with eating disorder [94]. Given the small amount of available data, more studies are needed to better understand the experience of parents/caregivers and the impact of treatments for them.

Despite several promising findings, what is striking from the current review is the relative lack of high-quality evidence and diversity within the available studies. Many studies, particularly those with uncontrolled designs, were small and likely underpowered. Of the seven RCT’s identified, three had less than 30 participants and failed to appropriately describe the randomisation process. Even the four larger RCT’s had relatively small sample sizes (n = 80–109), with one [21] acknowledging it was likely underpowered.

Variability in study setting was also observed. Nearly a quarter (4/18, 22.2%) of the included studies were conducted in a higher level of care (e.g. residential or day hospital setting) and several interventions [33, 36, 42] were delivered as adjuncts alongside standard care. Taken together, this makes it difficult to ascertain the contribution a specific intervention may be having on outcome. Follow-up reporting was also variable, with most studies only reporting end-of-treatment outcomes. As a result, outcomes at longer term follow-up cannot be determined with any confidence from the available data.

With regards to demographics, 97% of participants were female. Whilst prevalence rates are lower in males, it is estimated that BN affects 1% of men across their lifetime [2]. Additionally, most data available are for people from a White ethnic background. 65% of participants were identified as White, Caucasian or “non ethnic minority”. 33% of studies did not report ethnicity and only 17% reported on socioeconomic status. Lastly, the majority (56%) were conducted in the USA and none were conducted outside of the USA or Europe. While not unique to BN research, this limits the generalisability of the current findings to other countries and more diverse populations.

## Strengths and limitations

The current study has several strengths and limitations. The systematic methodology and broad remit are strengths of the review and allows for a comprehensive understanding of the available data. Inclusion of grey literature is also a strength.

Regarding limitations, the review was not preregistered and only English language papers were included, leading to the exclusion of 14 studies (see Fig. 1). While a broad scope of the literature was a strength of the study, it also increased the variability in outcomes measures, interventions reviewed, settings in which studies were conducted making interpretation and comparison between studies more challenging.

Intervention cost effectiveness and acceptability were not specifically explored but are important when considering real life clinical applicability. Similarly, predictor, moderator and mediator studies were beyond the scope of the current review.

## Future directions

Taken together, the current review provides several directions for future research. In addition to the need for more, higher quality, larger studies with more diverse samples, it would be useful for future studies to explore treatment mechanisms and how these target individual neurobiological and/or temperamental traits common in individuals with BN and other parent/carer factors. Little is currently understood about *how* and *why* current adolescent BN treatments work [95]. Predictor, moderator and mediator data offer some clues [96]. For example, Le Grange et al. [20] found that families presenting to treatment in their RCT with higher self-reported conflict within families had better outcomes in individual, rather than family-focused, treatment. Similarly, baseline eating disorder and depressive symptom severity, and early treatment response is associated with improved outcomes at end-of-treatment, and increased motivation to change may be associated with cognitive improvements [96]. This suggests different individuals and families may need differing interventions based on how they present to treatment.

Increasingly, studies are demonstrating a distinct cluster of traits associated with BN. There is a recognition that people with BN may present with lower impulse control [97], greater novelty seeking [98, 99], lower emotional awareness and clarity [100] and may be less motivated by future rewards [97], especially when compared to people with AN. There is also data to suggest food restriction, bingeing and purging may change brain structures [101]. It is also very common for people with BN to experience one or more mental health difficulties, including mood disorders, anxiety disorders, suicidal ideation, self-harm, and attention deficit hyperactivity disorder [5, 102–105]. In their study of 10,123 adolescents, Swanson et al. (2011) found that 88% of adolescents with BN met criteria for at least one other lifetime mental health disorder. Together, this suggest there may be distinct neuro-temperamental factors that are common for adolescents with BN, and if directly targeted, may improve outcomes. Pilot work in adolescent AN suggests targeting transdiagnostic traits may be helpful at improving outcomes and is perceived as helpful [106–108].

Little data currently exists to help elucidate whether treatments are targeting these factors effectively or not. Future adolescent BN studies that measure the impact of specific, theory-driven, treatment components [106] might help begin to answer this. Furthermore, studies that examine how these interact with common treatment factors (cf [107] for review of common factors research) and the context in which the treatment is delivered would also be useful.

In adolescent anorexia nervosa, qualitative data are beginning to emerge regarding perceived change mechanisms within family treatments [111]. Young people, parents and clinicians all describe the importance of a) a trusting, open relationships (therapeutic alliance), b) ensuring life outside the illness is part of treatment from the outset (holistic focus), and c) generating an environment in which the illness cannot be avoided [112–114]. Whether these apply to BN interventions or not is yet to be determined, however, understanding how people with BN experience change to occur within treatments is also important to consider.

## Conclusions

Whilst this review demonstrates the benefits of family-focused interventions, conclusions regarding its superiority above other interventions are tentative. This is predominantly due to the general underrepresentation of adolescent BN treatment studies in the literature. Studies are marred with likely selection bias, underpowering, uncontrolled or retrospective designs. The findings of this systematic scoping review reinforce the previously identified need for larger higher quality data across a range of intervention types; pharmacological and psychosocial. Future research that focuses on theory-driven mechanisms to target the broader presentation of BN are needed.

## Supplementary Information

Additional file 1.

Additional file 2.

Additional file 3.

## Data Availability

No datasets were generated or analysed during the current study.

## References

[1] Hail L, Le Grange D. Bulimia nervosa in adolescents: Prevalence and treatment challenges. Adolesc Health Med Ther. 2018;9:11–6. 29379324 10.2147/AHMT.S135326 PMC5757497

[2] van Eeden AE, van Hoeken D, Hoek HW. Incidence, prevalence and mortality of anorexia nervosa and bulimia nervosa. Curr Opin Psychiatry. 2021;34(6):515–24. 34419970 10.1097/YCO.0000000000000739 PMC8500372

[3] Piao J, Huang Y, Han C, Li Y, Xu Y, Liu Y, et al. Alarming changes in the global burden of mental disorders in children and adolescents from 1990 to 2019: a systematic analysis for the Global Burden of Disease study. Eur Child Adolesc Psychiatry. 2022;31(11):1827–45. 35831670 10.1007/s00787-022-02040-4

[4] Jones JM, Bennett S, Olmsted MP, Lawson ML, Rodin G. Disordered eating attitudes and behaviours in teenaged girls: a school-based study. Can Med Assoc Its Licens. 2001;165(5). PMC81412 11563206

[5] Swanson SA. Prevalence and correlates of eating disorders in adolescents: results from the national comorbidity survey replication adolescent supplement. Arch Gen Psychiatry. 2011;68(7):714. 21383252 10.1001/archgenpsychiatry.2011.22 PMC5546800

[6] Miniati M, Benvenuti A, Bologna E, Maglio A, Cotugno B, Massimetti G, et al. Mood spectrum comorbidity in patients with anorexia and bulimia nervosa. Eat Weight Disord Stud Anorex Bulim Obes. 2018;23(3):305–11. 10.1007/s40519-016-0333-1 27766498

[7] Fouladi F, Mitchell JE, Crosby RD, Engel SG, Crow S, Hill L, et al. Prevalence of alcohol and other substance use in patients with eating disorders: substance use in eating disorders. Eur Eat Disord Rev. 2015;23(6):531–6. 26415622 10.1002/erv.2410

[8] Makin L, Zesch E, Meyer A, Mondelli V, Tchanturia K. Autism, ADHD, and their traits in adults with bulimia nervosa and binge eating disorder: a scoping review. Eur Eat Disord Rev. 2025. 10.1002/erv.3177. 39865514 10.1002/erv.3177 PMC12171673

[9] Martinussen M, Friborg O, Schmierer P, Kaiser S, Øvergård KT, Neunhoeffer AL, et al. The comorbidity of personality disorders in eating disorders: a meta-analysis. Eat Weight Disord Stud Anorex Bulim Obes. 2017;22(2):201–9. 10.1007/s40519-016-0345-x 27995489

[10] Pearson CM, Lavender JM, Cao L, Wonderlich SA, Crosby RD, Engel SG, et al. Associations of borderline personality disorder traits with stressful events and emotional reactivity in women with bulimia nervosa. J Abnorm Psychol. 2017;126(5):531–9. 28691843 10.1037/abn0000225 PMC5505179

[11] Himmerich H, Hotopf M, Shetty H, Schmidt U, Treasure J, Hayes RD, et al. Psychiatric comorbidity as a risk factor for the mortality of people with bulimia nervosa. Soc Psychiatry Psychiatr Epidemiol. 2019;54(7):813–21. 30756148 10.1007/s00127-019-01667-0

[12] Slade E, Keeney E, Mavranezouli I, Dias S, Fou L, Stockton S, et al. Treatments for bulimia nervosa: a network meta-analysis. Psychol Med. 2018;48(16):2629–36. 29729686 10.1017/S0033291718001071

[13] Datta N, Matheson BE, Citron K, Van Wye EM, Lock JD. Evidence based update on psychosocial treatments for eating disorders in children and adolescents. J Clin Child Adolesc Psychol. 2023;52(2):159–70. 35950931 10.1080/15374416.2022.2109650

[14] Gorrell S, Le Grange D. Update on treatments for adolescent bulimia nervosa. Child Adolesc Psychiatr Clin N Am. 2019;28(4):537–47. 31443872 10.1016/j.chc.2019.05.002 PMC6709693

[15] National Institute for Health and Care Excellence (NICE). Eating Disorders (NICE Guideline ng69) [Internet]. 2017. Available from: Retrieved from: https://www.nice.org.uk/guidance/ng69. 39405396

[16] Hay PP, Bacaltchuk J, Stefano S. Psychotherapy for bulimia nervosa and bingeing. In: The Cochrane Collaboration, editor. Cochrane Database of Systematic Reviews [Internet]. Chichester, UK: John Wiley & Sons, Ltd; 2004 [cited 2025 Feb 9]. p. CD000562.pub2. Available from: 10.1002/14651858.CD000562.pub2. 10.1002/14651858.CD000562.pub2 15266434

[17] Le Grange D, Schmidt U. The treatment of adolescents with bulimia nervosa. J Ment Health. 2005;14(6):587–97.

[18] Kotler LA, Devlin MJ, Mark Davies B, Walsh T. An open trial of fluoxetine for adolescents with bulimia nervosa. J Child Adolescent Psychopharmacol. 2003;13(3):329–35. 10.1089/104454603322572660. 10.1089/104454603322572660 14642021

[19] Le Grange D, Crosby RD, Rathouz PJ, Leventhal BL. A randomized controlled comparison of family-based treatment and supportive psychotherapy for adolescent bulimia nervosa. Arch Gen Psychiatry. 2007;64(9):1049–56. 17768270 10.1001/archpsyc.64.9.1049

[20] Le Grange D, Lock J, Agras WS, Bryson SW, Jo B. Randomized clinical trial of family-based treatment and cognitive-behavioral therapy for adolescent bulimia nervosa. J Am Acad Child Adolesc Psychiatry. 2015;54(11):886-894.e2. 26506579 10.1016/j.jaac.2015.08.008 PMC4624104

[21] Schmidt U, Lee S, Beecham J, Perkins S, Treasure J, Yi I, et al. A randomized controlled trial of family therapy and cognitive behavior therapy guided self-care for adolescents with bulimia nervosa and related disorders. Am J Psychiatry. 2007;164(4):591–8. 17403972 10.1176/ajp.2007.164.4.591

[22] Stefini A, Salzer S, Reich G, Horn H, Winkelmann K, Bents H, et al. Cognitive-behavioral and psychodynamic therapy in female adolescents with bulimia nervosa: a randomized controlled trial. J Am Acad Child Adolesc Psychiatry. 2017;56(4):329–35. 28335877 10.1016/j.jaac.2017.01.019

[23] Peters MDJ, Godfrey CM, Khalil H, McInerney P, Parker D, Soares CB. Guidance for conducting systematic scoping reviews. Int J Evid Based Healthc. 2015;13(3):141–6. 26134548 10.1097/XEB.0000000000000050

[24] Page MJ, McKenzie JE, Bossuyt PM, Boutron I, Hoffmann TC, Mulrow CD, et al. The PRISMA 2020 statement: An updated guideline for reporting systematic reviews. J Clin Epidemiol. 2021;134:178–89. 33789819 10.1016/j.jclinepi.2021.03.001

[25] Munn Z, Peters MDJ, Stern C, Tufanaru C, McArthur A, Aromataris E. Systematic review or scoping review? Guidance for authors when choosing between a systematic or scoping review approach. BMC Med Res Methodol. 2018;18(1):143. 30453902 10.1186/s12874-018-0611-x PMC6245623

[26] Higgins JPT, Green S, editors. Cochrane Handbook for Systematic Reviews of Interventions (Version 5.1.0). The Cochrane Collaboration; 2013.

[27] Fahy T, Eisler I, Russell G. A placebo-controlled trial of d-fenfluramine in bulimia nervosa. Br J Psychiatry. 1993;162:597–603. 8149110 10.1192/bjp.162.5.597

[28] Knatz Peck S, Towne T, Wierenga CE, Hill L, Eisler I, Brown T, et al. Temperament-based treatment for young adults with eating disorders: acceptability and initial efficacy of an intensive, multi-family, parent-involved treatment. J Eat Disord. 2021;9(1):110. 34496951 10.1186/s40337-021-00465-x PMC8424819

[29] Nevonen L, Broberg A. A comparison of sequenced individual and group psychotherapy for patients with bulimia nervosa. Int J Eat Disord. 2006;39(2):117–27. 16231341 10.1002/eat.20206

[30] Sánchez-Ortiz VC, Munro C, Stahl D, House J, Startup H, Treasure J, et al. A randomized controlled trial of internet-based cognitive-behavioural therapy for bulimia nervosa or related disorders in a student population. Psychol Med. 2011;41(2):407–17. 20406523 10.1017/S0033291710000711

[31] Hong QN, Pluye P, Fabregues S, Bartlett G, Broardman F, Cargo M, et al. Mixed Method Appraisal Tool (MMAT) [Internet]. 2018. Available from: http://mixedmethodsappraisaltoolpublic.pbworks.com/.

[32] Peters MDJ, Godfrey CM, McInerney P, Munn Z, Tricco AC, Khalil AC. Chapter 11: Scoping Reviews (2020 version). In: Aromataris E, Munn Z, editors. JBI Manual for Evidence Synthesis, JBI, 2020 [Internet]. 2020. Available from: https://synthesismanual.jbi.global.

[33] Field T, Schanberg S, Kuhn C, Fierro K, Henteleff T, Mueller C, et al. Bulimic adolescents benefit from massage therapy. Adolescence. 1998;33(131):555–555. 9831872

[34] Johnson SM, Maddeaux C, Blouin J. Emotionally focused family therapy for bulimia: Changing attachment patterns. Psychotherapy. 1998;35(2):238–47.

[35] Wagner G, Nobis G, Mayerhofer A, Schau J, Spitzer M, Karwautz A, et al. Is technology assisted guided self-help successful in treating female adolescents with bulimia nervosa? Neuropsychiatrie. 2013;27(2):66–73. 23609487 10.1007/s40211-013-0062-x

[36] Lazaro L, Font E, Moreno E, Calvo R, Vila M, Andres-Perpina S, et al. Effectiveness of self-esteem and social skills group therapy in adolescent eating disorder patients attending a day hospital treatment programme. Eur Eat Disord Rev J Eat Disord Assoc. 2010;19(5):398–406. 10.1002/erv.1054 24081715

[37] Matheson BE, Bohon C, Le Grange D, Lock JD. Family-based treatment (FBT) for loss of control (LOC) eating in youth: current knowledge and future directions. Eat Disord. 2024;32(1):1–12. 38149636 10.1080/10640266.2023.2229091 PMC10753090

[38] Reilly EE, Gorrell S, Brosof L, Lock J, Le Grange D. Characterizing changes in obsessive–compulsive symptomsover the course of treatment for adolescent bulimia nervosa. Int J Eat Disord. 2022;55(10):1342–51. 35861249 10.1002/eat.23782 PMC9869712

[39] Valenzuela F, Lock J, Le Grange D, Bohon C. Comorbid depressive symptoms and self-esteem improve after either cognitive-behavioural therapy or family-based treatment for adolescent bulimia nervosa. Eur Eat Disord Rev. 2018;26(3):253–8. 29446174 10.1002/erv.2582 PMC6010314

[40] Dodge E, Hodes M, Eisler I, Dare C. Family therapy for bulimia nervosa in adolescents: an exploratory study. Spec Issue Eat Disord. 1995;17(1):59–77.

[41] Lebow J, Sim L, Wonderlich S, Peterson CB. Adapting integrative cognitive-affective therapy for adolescents with full and subthreshold bulimia nervosa: a feasibility study. Eur Eat Disord Rev. 2022;31:2946. 10.1002/erv.2946 35969509

[42] Martinez-Mallen E, Castro-Fornieles J, Lazaro L, Moreno E, Morer A, Font E, et al. Cue exposure in the treatment of resistant adolescent bulimia nervosa. Int J Eat Disord. 2007;40(7):596–601. 17607695 10.1002/eat.20423

[43] Murray SB, Anderson LK, Cusack A, Nakamura T, Rockwell R, Griffiths S, et al. Integrating family-based treatment and dialectical behavior therapy for adolescent bulimia nervosa: preliminary outcomes of an open pilot trial. Eat Disord. 2015;23(4):336–44. 26009971 10.1080/10640266.2015.1044345

[44] Pretorius N, Treasure J, Waller G, Yoshioka M, Schmidt U, Arcelus J, et al. Cognitive-behavioural therapy for adolescents with bulimic symptomatology: the acceptability and effectiveness of internet-based delivery. Behav Res Ther. 2009;47(9):729–36. 19515360 10.1016/j.brat.2009.05.006

[45] Stewart CS, Baudinet J, Hall R, Fiskå M, Pretorius N, Voulgari S, et al. Multi-family therapy for bulimia nervosa in adolescence: a pilot study in a community eating disorder service. Eat Disord. 2021;29(4):351–67. 31609163 10.1080/10640266.2019.1656461

[46] Le Grange D, Lock J. Treating bulimia in adolescents: a family-based approach. 1st ed. New York: Guilford Press; 2007. p. 260.

[47] Lock J. Adjusting cognitive behavior therapy for adolescents with bulimia nervosa: results of case series. Am J Psychother. 2005;59(3):267–81. 16370133 10.1176/appi.psychotherapy.2005.59.3.267

[48] Cooper Z, Fairburn C. The eating disorder examination: a semi-structured interview for the assessment of the specific psychopathology of eating disorders. Int J Eat Disord. 1987;6(1):1–8.

[49] Johnson C. Initial consultation for patients with bulimia and anorexia nervosa. In: Gamer DM, Garfinkel PE, editors. Handbook of psychotherapy for anorexia nervosa and bulimia. New York: Guilford; 1985. p. 19–51.

[50] Garner DM, Olmstead MP, Polivy J. Development and validation of a multidimensional eating disorder inventory for anorexia nervosa and bulimia. Int J Eat Disord. 1983;2(2):15–34.

[51] Blouin AG, Perez EL, Blouin JH. Computerized administration of the diagnostic interview schedule. Psychiatry Res. 1988;23(3):335–44. 3387505 10.1016/0165-1781(88)90024-8

[52] Hilbert A, Tuschen-Caffier B. Eating Disorder Examination-Questionnaire (Deutsche Version). Münst Verl Für Psychother. 2006.

[53] Carrard I, Rouget P, Fernández-Aranda F, Volkart AC, Damoiseau M, Lam T. Evaluation and deployment of evidence based patient self-management support program for bulimia nervosa. Int J Med Inf. 2006;75(1):101–9. 10.1016/j.ijmedinf.2005.07.031 16115793

[54] Kappel V, Thiel A, Holzhausen M, Jaite C, Schneider N, Pfeiffer E, et al. Eating Disorder Inventory (EDI-2): Normative data among 10 to 20 year old German girls and boys. Eat Disord Inventory-2 EDI-2 Normierung Einer Stichprobe Norm Sch Im Alter Von 10 Bis 20 Jahren Patientinnen Mit Anorex Nerv. 2012; 58(3):127–44.

[55] Lebow J, Sim L, Wonderlich S, Peterson CB. Adapting integrative cognitive-affective therapy for adolescents with full and subthreshold bulimia nervosa: a feasibility study. Eur Eat Disord Rev. 2023;31(1):178–87. 35969509 10.1002/erv.2946

[56] Russell GFM, Szmukler GI, Dare C, Eisler I. An evaluation of family therapy in anorexia nervosa and bulimia nervosa. Arch Gen Psychiatry. 1987;44(12):1047. 3318754 10.1001/archpsyc.1987.01800240021004

[57] Garner DM, Garfinkel PE. The eating attitudes test: an index of the symptoms of anorexia nervosa. Psychol Med. 1979;9(2):273–9. 472072 10.1017/s0033291700030762

[58] Baudinet J, Eisler I, Dawson L, Simic M, Schmidt U. Multi-family therapy for eating disorders: a systematic scoping review of the quantitative and qualitative findings. Int J Eat Disord. 2021;54(12):2095–120. 34672007 10.1002/eat.23616 PMC9298280

[59] Baudinet J, Eisler I. Multi-family therapy for eating disorders across the lifespan. Curr Psychiatry Rep. 2024. 10.1007/s11920-024-01504-5. 38709444 10.1007/s11920-024-01504-5 PMC11147926

[60] Fairburn CG, Beglin SJ. Assessment of eating disorders: Interview or self-report questionnaire? Int J Eat Disord. 1994;16(4):363–70. 7866415

[61] Anderson LK, Murray SB, Ramirez AL, Rockwell R, Le Grange D, Kaye WH. The integration of family-based treatment and dialectical behavior therapy for adolescent bulimia nervosa: philosophical and practical considerations. Eat Disord. 2015;23(4):325–35. 26009868 10.1080/10640266.2015.1042319

[62] Thelen MH, Farmer J, Wonderlich S, Smith M. A revision of the bulimia test: the BULIT-R. Assess J Consult Clin Psychol. 1991;3(1):119–24.

[63] Garner DM, Olmsted MP, Bohr Y, Garfinkel PE. The eating attitudes test: psychometric features and clinical correlates. Psychol Med. 1982;12(4):871–8. 6961471 10.1017/s0033291700049163

[64] National Institute of Mental Health. Rating scales and assessment instruments for use in pediatric psychopharmacology research. Psychopharmacol Bull. 1985;21:839–43. 3911249

[65] Cooper PJ, Taylor M, Cooper Z, Fairburn CG. The development and validation of the body shape questionnaire. Int J Eat Disord. 1987;6(4):485–94.

[66] Derogatis LR. SCL-90-R: Administration, Scoring & Procedures Manual-II, for the R (Revised) Version and Other Instruments of the Psychopathology Rating Scale Series (2nd Edition). Clin Psychom Res Inc Towson. 1992;

[67] Beck AT, Steer RA, Carbin MG. Psychometric properties of the beck depression inventory: twenty-five years of evaluation. Clin Psychol Rev. 1988;8(1):77–100.

[68] McNair DM, Lorr M, Droppleman LF. Profile of Mood States (POMS). San Diego, CA: Educational and Industrial Testing Service; 1971.

[69] Spielberger C, Gorsuch R, Lushene R. STAI manual for the state trait anxiety inventory. Palo Alto: Consulting Psychologists Press; 1970.

[70] Field T, Morrow C, Valdeon C, Larson S, Kuhn C, Schanberg S. Massage reduces anxiety in child and adolescent psychiatric patients. J Am Acad Child Adolesc Psychiatry. 1992;31(1):125–31. 1537763 10.1097/00004583-199201000-00019

[71] Radloff LS. The CES-D Scale: a self-report depression scale for research in the general population. Appl Psychol Meas. 1977;1(3):285–401.

[72] Beck AT, Steer RA, Brown GK. Manual for the Beck Depression Inventory-II. San Antonio: Psychological Corporation; 1996.

[73] Rosenberg M. Society and the adolescent self-image. Princeton: Princeton University Press; 1965.

[74] Scahill L, Riddle MA, McSWIGGIN-HARDIN M, Ort SI, King RA, Goodman WK, et al. Children’s yale-brown obsessive compulsive scale: reliability and validity. J Am Acad Child Adolesc Psychiatry. 1997;36(6):844–52. 9183141 10.1097/00004583-199706000-00023

[75] Bellace DL, Tesser R, Berthod S, Wisotzke K, Crosby RD, Crow SJ, et al. The yale-brown-cornell eating disorders scale self-report questionnaire: a new, efficient tool for clinicians and researchers. Int J Eat Disord. 2012;45(7):856–60. 22532411 10.1002/eat.22023 PMC5582801

[76] Chorpita BF, Moffitt CE, Gray J. Psychometric properties of the revised child anxiety and depression scale in a clinical sample. Behav Res Ther. 2005;43(3):309–22. 15680928 10.1016/j.brat.2004.02.004

[77] Gratz KL, Roemer L. Multidimensional assessment of emotion regulation and dysregulation: development, factor structure, and initial validation of the difficulties in emotion regulation scale. J Psychopathol Behav Assess. 2004;26(1):41–54.

[78] Piers EV, Harris DB. The Piers–Harris Children’s Self-Concept Scale. Nashville, Tennesse: Counselor Recording and Tests. 1969.

[79] Gila A, Castro J, Gómez MJ, Toro J. Social and body self-esteem in adolescents with eating disorders. Int J Psychol Psycholgical Ther. 2005;1(1):63–71.

[80] Silva F, Martorell MC. Bateria de Socializacio ´n BAS-3. Manual. Madrid: TEA Ediciones, S.A.; 1989.

[81] Birmaher B, Brent DA, Chiappetta L, Bridge J, Monga S, Baugher M. Psychometric properties of the screen for child anxiety related emotional disorders (SCARED): a replication study. J Am Acad Child Adolesc Psychiatry. 1999;38(10):1230–6. 10517055 10.1097/00004583-199910000-00011

[82] Rhodes P, Baillie A, Brown J, Madden S. Parental efficacy in the family-based treatment of anorexia: preliminary development of the Parents Versus Anorexia Scale (PVA). Eur Eat Disord Rev. 2005;13(6):399–405.

[83] Zigmond AS, Snaith RP. The hospital anxiety and depression scale. Acta Psychiatr Scand. 1983;67(6):361–70. 6880820 10.1111/j.1600-0447.1983.tb09716.x

[84] Szmukler GI, Burgess P, Herrman H, Bloch S, Benson A, Colusa S. Caring for relatives with serious mental illness: the development of the Experience of Caregiving Inventory. Soc Psychiatry Psychiatr Epidemiol. 1996;31(3–4):137–48. 8766459 10.1007/BF00785760

[85] Shapiro JR, Berkman ND, Brownley KA, Sedway JA, Lohr KN, Bulik CM. Bulimia nervosa treatment: a systematic review of randomized controlled trials. Int J Eat Disord. 2007;40(4):321–36. 17370288 10.1002/eat.20372

[86] Agras WS, Walsh BT, Fairburn CG, Wilson GT, Kraemer HC. A multicenter comparison of cognitive-behavioral therapy and interpersonal psychotherapy for Bulimia Nervosa. Arch Gen Psychiatry. 2000;57(5):459. 10807486 10.1001/archpsyc.57.5.459

[87] Poulsen S, Lunn S, Daniel SIF, Folke S, Mathiesen BB, Katznelson H, Fairburn CG. A randomized controlled trial of psychoanalytic psychotherapy or cognitive-behavioral therapy for bulimia nervosa. Am J Psychiatry. 2014;171(1):109–16. 10.1176/appi.ajp.2013.12121511. 24275909 10.1176/appi.ajp.2013.12121511

[88] Austin A, Anderson AG, Lee J, Vander Steen H, Savard C, Bergmann C, Singh M, Devoe D, Gorrell S, Patten S, Le Grange D, Dimitropoulos G. Efficacy of eating disorder focused family therapy for adolescents with anorexia nervosa: a systematic review and meta‐analysis. Int J Eati Disord. 2024;58(1):3–36. 10.1002/eat.24252. 10.1002/eat.24252 PMC11754536 39041682

[89] Hilbert A, Hoek HW, Schmidt R. Evidence-based clinical guidelines for eating disorders: international comparison. Curr Opin Psychiatry. 2017;30(6):423–37. 28777107 10.1097/YCO.0000000000000360 PMC5690314

[90] Maddocks SE, Kaplan AS, Woodside DB, Langdon L, Piran N. Two year follow-up of bulimia nervosa: the importance of abstinence as the criterion of outcome. Int J Eat Disord. 1992;12(2):133–41.

[91] Hay P. A systematic review of evidence for psychological treatments in eating disorders: 2005–2012. Int J Eat Disord. 2013;46(5):462–9. 23658093 10.1002/eat.22103

[92] Escoffié A, Pretorius N, Baudinet J. Multi-family therapy for bulimia nervosa: a qualitative pilot study of adolescent and family members’ experiences. J Eat Disord. 2022;10(1):91. 35786421 10.1186/s40337-022-00606-w PMC9250718

[93] Baudinet J, Simic M. Adolescent eating disorder day programme treatment models and outcomes: a systematic scoping review. Front Psychiatry. 2021;29(12):539. 10.3389/fpsyt.2021.652604 PMC8116630 33995149

[94] Zabala MJ, Macdonald P, Treasure J. Appraisal of caregiving burden, expressed emotion and psychological distress in families of people with eating disorders: a systematic review. Eur Eat Disord Rev. 2009;17(5):338–49. 19367608 10.1002/erv.925

[95] Baudinet J, Eisler I. Introduction to evidence-based psychological approaches in the treatment of eating disorders: what is the evidence in evidence based practice? In: Robinson P, Wade T, Herpertz-Dahlmann B, Fernandez-Aranda F, Treasure J, Wonderlich S, editors. Eating disorders: an international comprehensive view. Cham: Springer; 2023. p. 1–21. 10.1007/978-3-030-97416-9_99-1.

[96] Gorrell S, Byrne CE, Trojanowski PJ, Fischer S, Le Grange D. A scoping review of non-specific predictors, moderators, and mediators of family-based treatment for adolescent anorexia and bulimia nervosa: a summary of the current research findings. Eat Weight Disord Stud Anorex Bulim Obes. 2022;27(6):1971–90. 10.1007/s40519-022-01367-w PMC9872820 35092554

[97] Wierenga CE, Ely A, Bischoff-Grethe A, Bailer UF, Simmons AN, Kaye WH. Are extremes of consumption in eating disorders related to an altered balance between reward and inhibition? Front Behav Neurosc. 2014. 10.3389/fnbeh.2014.00410/abstract. 10.3389/fnbeh.2014.00410 PMC4260511 25538579

[98] Atiye M, Miettunen J, Raevuori-Helkamaa A. A meta-analysis of temperament in eating disorders: temperament in eating disorders. Eur Eat Disord Rev. 2015;23(2):89–99. 25546554 10.1002/erv.2342

[99] Rotella F, Mannucci E, Gemignani S, Lazzeretti L, Fioravanti G, Ricca V. Emotional eating and temperamental traits in Eating Disorders: a dimensional approach. Psychiatry Res. 2018;264:1–8. 29626825 10.1016/j.psychres.2018.03.066

[100] Weinbach N, Sher H, Bohon C. Differences in emotion regulation difficulties across types of eating disorders during adolescence. J Abnorm Child Psychol. 2018;46(6):1351–8. 29101588 10.1007/s10802-017-0365-7 PMC6014925

[101] Frank GKW, Shott ME, DeGuzman MC. The Neurobiology of Eating Disorders. Child Adolesc Psychiatr Clin N Am. 2019;28(4):629–40. 31443880 10.1016/j.chc.2019.05.007 PMC6709695

[102] Blinder BJ, Cumella EJ, Sanathara VA. Psychiatric Comorbidities of Female Inpatients With Eating Disorders. Psychosom Med [Internet]. 2006;68(3). Available from: https://journals.lww.com/psychosomaticmedicine/Fulltext/2006/05000/Psychiatric_Comorbidities_of_Female_Inpatients.16.aspx. 10.1097/01.psy.0000221254.77675.f5 16738079

[103] Hovrud L, Simons R, Simons J, Korkow J. Non-suicidal self-injury and bulimia: the role of emotion dysregulation and body dissatisfaction. Eat Weight Disord Stud Anorex Bulim Obes. 2020;25(4):1089–97. 10.1007/s40519-019-00741-5 PMC7399667 31292855

[104] Lavender JM, Wonderlich SA, Peterson CB, Crosby RD, Engel SG, Mitchell JE, et al. Dimensions of emotion dysregulation in bulimia nervosa: emotion dysregulation dimensions in BN. Eur Eat Disord Rev. 2014;22(3):212–6. 24619484 10.1002/erv.2288 PMC4554700

[105] Seitz J, Kahraman-Lanzerath B, Legenbauer T, Sarrar L, Herpertz S, Salbach-Andrae H, et al. The role of impulsivity, inattention and comorbid ADHD in patients with Bulimia Nervosa. PLoS ONE. 2013;8(5):e63891. 23700439 10.1371/journal.pone.0063891 PMC3659086

[106] Baudinet J, Stewart C, Bennett E, Konstantellou A, Parham R, Smith K, et al. Radically open dialectical behaviour therapy adapted for adolescents: a case series. BMC Psychiatry. 2021;21(1):462. 34551741 10.1186/s12888-021-03460-3 PMC8456700

[107] Baudinet J, Simic M, Griffiths H, Donnelly C, Stewart C, Goddard E. Targeting maladaptive overcontrol with radically open dialectical behaviour therapy in a day programme for adolescents with restrictive eating disorders: an uncontrolled case series. J Eat Disord. 2020;8(1):68. 33292696 10.1186/s40337-020-00338-9 PMC7663904

[108] Baudinet J, Watson C, Brothwood PL, Parham R, Smith L, Snowden N, et al. Adolescent experience of radically open dialectical behaviour therapy: a qualitative study. BMC Psychiatry. 2022;22(1):466. 35836210 10.1186/s12888-022-04114-8 PMC9281135

[109] Michie S, Van Stralen MM, West R. The behaviour change wheel: A new method for characterising and designing behaviour change interventions. Implement Sci. 2011;6(1):42. 21513547 10.1186/1748-5908-6-42 PMC3096582

[110] Cuijpers P, Reijnders M, Huibers MJH. The role of common factors in psychotherapy outcomes. Annu Rev Clin Psychol. 2019;15(1):207–31. 30550721 10.1146/annurev-clinpsy-050718-095424

[111] Cripps S, Serpell L, Pugh M. Processes of change in family therapies for anorexia nervosa: a systematic review and meta-synthesis of qualitative data. J Eat Disord. 2024;12(1):104. 39054560 10.1186/s40337-024-01037-5 PMC11270895

[112] Baudinet J, Eisler I, Konstantellou A, Simic M, Schmidt U. How young people perceive change to occur in family therapy for anorexia nervosa: a qualitative study. J Eat Disord. 2024;12(1):11. 38254187 10.1186/s40337-024-00971-8 PMC10804743

[113] Baudinet J, Eisler I, Roddy M, Turner J, Simic M, Schmidt U. Clinician perspectives on how change occurs in multi-family therapy for adolescent anorexia nervosa: a qualitative study. J Eat Disord. 2024;12(1):103. 39049063 10.1186/s40337-024-01064-2 PMC11267764

[114] Baudinet J, Eisler I, Konstantellou A, Hunt T, Kassamali F, McLaughlin N, et al. Perceived change mechanisms in multi-family therapy for anorexia nervosa: a qualitative follow-up study of adolescent and parent experiences. Eur Eat Disord Rev. 2023;31(6):822–36. 37415392 10.1002/erv.3006

[115] Baudinet J, Simic M. Integrated family therapy for adolescent bulimia nervosa: a treatment manual. In preparation. London, UK: Routledge; 2025.

