# Supplementary material for: Treatment outcomes for adolescent bulimia nervosa: a systematic scoping review of quantitative findings
Source: J Eat Disord. 2025 Apr 16;13:68. doi: 10.1186/s40337-025-01236-8 (PMC12004555; doi:10.1186/s40337-025-01236-8)
Supplement: Supplementary file 1 — Additional file 1. [file 40337_2025_1236_MOESM1_ESM.pdf]

Supplementary Material 1: Search Terms and Strategy

Main databases: Medline, PsychInfo, Embase, CENTRAL, CINAHL
Search Date: 4th September 2024

Concept & Terms		Databases PsychInfo, Medline, Embase (Ovid)	Database CENTRAL (Cochrane)
**Concept: Adolescent**			
Adolescent/Adolescence	1	Adolescen*	Adolescen*
Child/Children	2	Child*	Child*
Puberty/Pubertal	3	Pubert*	Pubert*
Teen/teenager/teenage	4	Teen*	Teen*
	5	OR/1-4	{OR #1- #4}
**Concept: Bulimia Nervosa**			
MeSH Term	6	*Bulimia Nervosa/cl, di [Classification, Diagnosis]	[Bulimia Nervosa] explode all trees
Bulimia Nervosa	7	Bulimia Nervosa	"Bulimia Nervosa"
Bulimia Nervosa	8	Bulimi*	Bulimi*
	9	OR/6-8	{OR #6- #8}
**Concept: Intervention**			
Therapy/Therapeutic	10	Therap*	Therap*
Psychology/Psychologie	11	Psycholog*	Psycholog*
Psychodynamic psychotherapy	12	Psychodynami* psychotherap*	Psychodynami* psychotherap*
Psychodrama	13	Psychodrama*	Psychodrama*
Psychoanalytic/Psychoanalysis	14	Psychoanaly*	Psychoanaly*
Psychotherapy/Psychotherapeutic	15	Psychotherap*	Psychotherap*
Psychoeducation	16	Psychoeducatio*	Psychoeducatio*
Behavioral Therapy/Behavioural Therapy/Behaviour therapy/Behavior Therapy	17	Behavi?* Therap*	Behavi* Therap*
Cognitive Therapy	18	Cognitive Therap*	Cognitive Therap*
Acceptance and commitment therapy	19	Acceptance and commitment therapy	"Acceptance and commitment therapy"
Compassion focused therapy/ Compassion focussed therapy	20	Compassion focus?ed therap*	"compassion focused therapy"
Abbreviation: CFT	21	CFT	CFT
Cognitive analytic therapy	22	Cognitive analyti* therap*	Cognitive analyti* therap*
Cognitive behavioural therapy	23	Cognitive behavio?ral therap*	Cognitive behavio* therap*
Abbreviation: CBT	24	CBT	CBT
Mindfulness based therapy	25	Mindfulne* based therap*	Mindfulne* based therap*
Abbreviation: MBT	26	MBT	MBT
Dialectical Behavioral therapy/ Dialectical Behavior therapy/ Dialectical behavioural therapy/ Dialectical behaviour therapy	27	Dialectical Behavio?* therap*	Dialectical Behavio* therap*
Abbreviation: DBT	28	DBT	DBT
Schema therapy	29	Schema therap*	Schema therap*
Family therapy	30	Famil* therap*	Famil* therap*
Family based therapy	31	Family based therapy	"Family based therapy"
Art therapy	32	Art* Therap*	Art* Therap*
Drama therapy	33	Drama* therap*	Drama* therap*
Dance movement psychotherapy	34	Dance movement psychotherap*	"Dance movement psychotherapy"
Computer Assisted therapy	35	Computer Assist* therap*	Computer Assist* therap*
Group Therapy	36	Group Therap*	Group Therap*
Diet therapy	37	Diet therap*	"Diet therapy"
Cue Exposure	38	Cue Exposure	"Cue Exposure"
Day Care	39	Day Care	"Day Care"
Day Programme/ Day Program	40	Day Program?*	Day Progra*
Psychopharmacology/ies	41	Psychopharmacolog*	Psychopharmacolog*
MESH term	42	Antidepressive agents/ or antidepressive agents, second-generation/ or antidepressive agents, tricyclic/	[Antidepressive Agents] explode all trees
Antidepressive agents/antidepressant	43	Antidepress*	Antidepress*
Fluoxetine	44	Fluoxetine	Fluoxetine
SSRI	45	SSRI	SSRI
Selective serotonin reuptake inhibitor	46	Selective serotonin reuptake inhibitor	"Selective serotonin reuptake inhibitor"
Serotonin uptake inhibitors	47	Serotonin uptake inhibitors	"Serotonin uptake inhibitors"
Antipsychotic	48	Antipsychotic*	Antipsychotic*
Mood stabilizer/stabiliser	49	Mood stabili?er*	Mood stabili*
Tricyclic	50	Tri?cyclic*	Tricyclic*

TCA	51	TCA	TCA
Mono amine oxidase inhibitor	52	Mono amine oxidase inhibitor*	“Mono amine oxidase inhibitor”
MAOI	53	MAOI	MAOI
Counselling	54	Counselling	Counselling
Self help/ self-help	55	Self help	“Self help”
Clinical trial	57	Clinical trial	“Clinical Trial”
Randomised control trial/ Randomized control trial	58	Randomi?ed control trial	Randomi* control trial
Randomized clinical trial/ Randomised clinical trial	59	Randomi?ed clinical trial	Randomi* clinical trial
Abbreviation: RCT	60	RCT	RCT
Observational study/studies	61	Observational stud*	Observational stud*
Case series	62	Case series	“Case series”
Cohort study	63	Cohort stud*	Cohort stud*
Uncontrolled study	64	Uncontrolled stud*	Uncontrolled stud*
Case control study	65	Case control stud*	Case control stud*
Family based treatment	66	Family based treatment	“Family based treatment”
Acronym	67	FBT	FBT
	68	Or/10-67	{OR #10- #67}
Search		#5 AND #9 AND #68	

CINAHL	TI (Adolescen* OR Child* OR Pubert* OR Teen*) AND TI ("Bulimia Nervosa" OR Bulimi*) AND TI (Therap* OR psycholog* OR "Psychodynami* psychotherap*" OR psychodrama* OR psychoanaly* OR psychotherap* OR psychoeducatio* OR "Behavi* Therap*" OR "Cognitive Therap*" OR "Acceptance and commitment therapy" OR "compassion focused therapy" OR cft OR "Cognitive analyti* therap*" OR "Cognitive behavio* therap*" OR cbt OR "Mindfulne* based therap*" OR mbt OR "Dialectical Behavio* therap*" OR dbt OR "Schema therap*" OR "Famil* therap*" OR "Family based therapy" OR "Art* Therap*" OR "Drama* therap*" OR "Dance movement psychotherapy" OR "Computer Assist* therap*" OR "Group Therap*" OR "Diet therapy" OR "Cue Exposure" OR "Day Care" OR "Day Progra*" OR psychopharmacolog* OR antidepress* OR fluoxetine OR ssri OR "Selective serotonin reuptake inhibitor" OR "Serotonin uptake inhibitors" OR "Antipsychotic*" OR "Mood stabili*" OR tricyclic* OR tca OR "Mono amine oxidase inhibitor" OR maoi OR counselling OR "Self help" OR "Clinical Trial" OR "Randomi* control trial" OR "Randomi* clinical trial" OR rct OR "Observational stud*" OR "Case series" OR "Cohort stud*" OR "Uncontrolled stud*" OR "Case control stud*" OR "Family based treatment" OR fbt)

Grey Literature Databases: SCOPUS, Web of Science, ProQuest Dissertations and Theses Global
Search Date: 14th March 2023

Database	Search Terms
SCOPUS	TITLE-ABS (adolescen* OR child* OR pubert* OR teen*) AND TITLE-ABS ("Bulimia Nervosa" OR bulimi*) AND TITLE-ABS (therap* OR psycholog* OR "Psychodynami*psychotherap*" OR psychodrama* OR psychoanaly* OR psychotherap* OR psychoeducatio* OR "Behavi* Therap*" OR "Cognitive Therap*" OR "Acceptance and commitment therapy" OR "compassion focused therapy" OR cft OR "Cognitive analyti* therap*" OR "Cognitive behavio* therap*" OR cbt OR "Mindfulne* based therap*" OR mbt OR "Dialectical Behavio* therap*" OR dbt OR "Schema therap*" OR "Famil* therap*" OR "Family based therapy" OR "Art* Therap*" OR "Drama* therap*" OR "Dance movement psychotherapy" OR "Computer Assist* therap*" OR "Group Therap*" OR "Diet therapy" OR "Cue Exposure" OR "Day Care" OR "Day Progra*" OR psychopharmacolog* OR antidepress* OR fluoxetine OR ssri OR "Selective serotonin reuptake inhibitor" OR "Serotonin uptake inhibitors" OR "Antipsychotic*" OR "Mood stabili*" OR tricyclic* OR tca OR "Mono amine oxidase inhibitor" OR maoi OR counselling OR "Self help" OR "Clinical Trial" OR "Randomi* control trial" OR "Randomi* clinical trial" OR rct OR "Observational stud*" OR "Case series" OR "Cohort stud*" OR "Uncontrolled stud*" OR "Case control stud*" OR "Family based treatment" OR fbt) AND (LIMIT-TO (LANGUAGE , "English"))
Web of Science	(TS=(Adolescen*) OR TS=(Child*) OR TS=(Pubert*) OR TS=(Teen*)) AND (TS=(“Bulimia Nervosa”) OR TS=(Bulimi*)) AND (TS=(Therap*) OR TS=(Psycholog*) OR TS=("Psychodynami* psychotherap*") OR TS=(Psychodrama*) OR TS=(Psychoanaly*) OR TS=(“Psychotherap*”) OR TS=(Psychoeducatio*) OR TS=(“Behavi* Therap*”) OR TS=(“Cognitive Therap*”) OR TS=(“Acceptance and commitment therapy”) OR TS=(“compassion focused therapy”) OR TS=(CFT) OR TS=(“Cognitive analyti* therap*”) OR TS=(“Cognitive behavio* therap*”) OR TS=(CBT) OR TS=(“Mindfulne* based therap*”) OR TS=(MBT) OR TS=(“Dialectical Behavio* therap*”) OR TS=(DBT) OR TS=(“Schema therap*”) OR TS=(“Famil* therap*”) OR TS=(“Family

	based therapy") OR TS=("Art* Therap*") OR TS=("Drama* therap*") OR TS=("Dance movement psychotherapy") OR TS=("Computer Assist* therap*") OR TS=("Group Therap*") OR TS=("Diet therapy") OR TS=("Cue Exposure") OR TS=("Day Care") OR TS=("Day Progra*") OR TS=(Psychopharmacolog*) OR TS=(Antidepress*) OR TS=(Fluoxetine) OR TS=(SSRI) OR TS=("Selective serotonin reuptake inhibitor") OR TS=("Serotonin uptake inhibitors") OR TS=(Antipsychotic*) OR TS=("Mood stabili*") OR TS=(Tricyclic*) OR TS=(TCA) OR TS=("Mono amine oxidase inhibitor") OR TS=(MAOI) OR TS=(Counselling) OR TS=("Self help") OR TS=("Clinical Trial") OR TS=("Randomi* control trial") OR TS=("Randomi* clinical trial") OR TS=(RCT) OR TS=("Observational stud*") OR TS=("Case series") OR TS=("Cohort stud*") OR TS=("Uncontrolled stud*") OR TS=("Case control stud*") OR TS=("Family based treatment") OR TS=(FBT))
ProQuest	ti((Adolescen* OR Child* OR Pubert* OR Teen*)) AND ti(("Bulimia Nervosa" OR Bulimi*)) AND ti((Therap* OR psycholog* OR "Psychodynami* psychotherap*" OR psychodrama* OR psychoanaly* OR psychotherap* OR psychoeducatio* OR "Behavi* Therap*" OR "Cognitive Therap*" OR "Acceptance and commitment therapy" OR "compassion focused therapy" OR cft OR "Cognitive analyti* therap*" OR "Cognitive behavio* therap*" OR cbt OR "Mindfulne* based therap*" OR mbt OR "Dialectical Behavio* therap*" OR dbt OR "Schema therap*" OR "Famil* therap*" OR "Family based therapy" OR "Art* Therap*" OR "Drama* therap*" OR "Dance movement psychotherapy" OR "Computer Assist* therap*" OR "Group Therap*" OR "Diet therapy" OR "Cue Exposure" OR "Day Care" OR "Day Progra*" OR psychopharmacolog* OR antidepress* OR fluoxetine OR ssri OR "Selective serotonin reuptake inhibitor" OR "Serotonin uptake inhibitors" OR "Antipsychotic*" OR "Mood stabili*" OR tricyclic* OR tca OR "Mono amine oxidase inhibitor" OR maoi OR counselling OR "Self help" OR "Clinical Trial" OR "Randomi* control trial" OR "Randomi* clinical trial" OR rct OR "Observational stud*" OR "Case series" OR "Cohort stud*" OR "Uncontrolled stud*" OR "Case control stud*" OR "Family based treatment" OR fbt))
