# Supplementary material for: Treatment outcomes for adolescent bulimia nervosa: a systematic scoping review of quantitative findings
Source: J Eat Disord. 2025 Apr 16;13:68. doi: 10.1186/s40337-025-01236-8 (PMC12004555; doi:10.1186/s40337-025-01236-8)
Supplement: Supplementary file 2 — Additional file 2. [file 40337_2025_1236_MOESM2_ESM.pdf]

Supplementary Material 2: **Mixed Method Appraisal Tool (MMAT)** (Hong et al., 2018)

- Reviewer: Dr Madeleine Love
- Date: 10.09.2024
- Papers Screened**:** 18

Paper	Author	Year	Number of included participants	Study Design
1	Dodge et al	1995	8	Single-arm study (Case series)
2	Field et al	1998	24	RCT
3	Johnson et al	1998	13	RCT
4	Kotler et al	2003	13	Single-arm study (Case series)
5	Lazaro et al	2011	44	Single-arm study (Case series)
6	Lebow et al	2023	8	Single-arm study (Case series)
7	Le Grange et al	2007	80	RCT
8	Le Grange et al	2015	109	RCT
9	Martinez-Mallen et al	2007	25	Single-arm study (Case series)
10	Matheson et al	2024	51* Excluded from total	RCT (Secondary Analysis Le Grange 2015)
11	Murray et al	2015	40	Single-arm study (Case series)
12	Pretorius et al	2009	101	Single-arm study (Case series)
13	Reilly et al	2022	109 *Excluded from total	RCT (Secondary Analysis Le Grange 2015)
14	Schmidt et al	2007	85	RCT
15	Stefini et al	2017	81	RCT
16	Stewart et al	2021	50	Single-arm study (Retrospective audit)
17	Valenzuela et al	2018	109 *Excluded from total	RCT (Secondary Analysis Le Grange 2015)
18	Wagner et al	2013	29	RCT
**Total Number of Participants included**			**710**	

Part I: Mixed Methods Appraisal Tool (MMAT) version 2018

- Screening Question 1 (S1): Are there clear research questions?
- Screening Question 2 (S2): Do the collected data allow to address the research question?

Answer options: Yes (Y), No (N), Can't tell (CT), Comments

	1	2	3	4	5	6	7	8	9	10	11	12	13	14	15	16	17	18
S1	Y	Y	Y	Y	Y	Y	Y	Y	Y	Y	Y	Y	Y	Y	Y	Y	Y	Y
S2	Y	Y	Y	Y	Y	Y	Y	Y	Y	Y	Y	Y	Y	Y	Y	Y	Y	Y

1. **Qualitative (n=0)**
2. **Quantitative Randomized Controlled Trial (n=7)**

- 2.1: Is randomization appropriately performed?
- 2.2: Are the groups comparable at baseline?
- 2.3: Are there complete outcome data? *Study authors agreed complete outcome data to be represented by 80% completion rate*
- 2.4: Are outcome assessors blinded to the intervention provided?
- 2.5: Did the participants adhere to the assigned intervention?

Answer options: Yes (Y), No (N), Can't tell (CT)

Primary RCTs (n=7)

	2	3	7	8	14	15	18
2.1	CT	CT	Y	Y	Y	Y	CT
2.2	CT	CT	Y	Y	Y	Y	CT
2.3	CT	CT	Y	Y	Y	Y	CT
2.4	CT	CT	N	Y	N	N	CT
2.5	CT	CT	Y	Y	Y	Y	N

Comments:

Paper 2 (Field et al., 1998) – Criteria complete 0%: Outcome – **LOW QUALITY**

- 2.1 **Can't Tell** Paper methodology reports "twenty-four female adolescent bulimic inpatients were randomly assigned to a massage therapy or standard treatment (control) group" hence inclusion under RCT study design. However, did not comment further on the randomisation process.
- 2.2 **Can't Tell** Paper does not comment on group demographics or whether statistical analysis was used to compare these
- 2.3 **Can't Tell** Not specifically stated whether all participants completed intervention
- 2.4 **Can't Tell** Paper methodology did not comment on blinding
- 2.5 **Can't Tell** Did not specifically state whether all participants adhered to assigned intervention

Paper 3 (Johnson et al., 1998) – Criteria complete 0%: Outcome **LOW QUALITY**

- 2.1 **Can't Tell** Paper methodology reports "participants were randomly assigned to EFFT or a cognitive behavioural education group" hence inclusion under RCT study design. However, did not comment further on the randomisation process.
- 2.2 **Can't Tell** Paper does not comment on group demographics or whether statistical analysis used to compare these
- 2.3 **Can't Tell** Not specifically stated whether all participants completed intervention and contributed to outcome data. Did however comment that one family dropped out of EFFT after session 1.
- 2.4 **Can't Tell** Paper methodology did not comment on blinding
- 2.5 **Can't Tell** Did not specifically state whether all participants adhered to assigned intervention, however did comment that one family dropped out of EFFT after session 1.

Paper 7 (Le Grange et al., 2007) – Criteria complete 80%: Outcome **MODERATE QUALITY**

- 2.1 **Yes** Randomisation was performed by an independent bio-statistician and was stratified in blocks of 4 or 6 for each therapist and each participant (i.e. at initial assessment, qualifying participants then divided into 2 groups, full BN and partial BN, and then assigned a therapist). After this assignment, participants were randomized to 1 or 2 treatment conditions. Participants, study staff, and therapists were, therefore, unable to predict treatment assignment of the next subject in the stream.
- 2.2 **Yes** Treatment groups were compared on sociodemographic and clinical characteristics at baselines using the Fisher exact test for dichotomous variables (e.g. sex and family status), the X^2 test for categorical variables (e.g. ethnicity and comorbid diagnoses) and independent sample *t* tests for continuous measures (e.g. age and BMI)
- 2.3 **Yes** 41/41 and 39/39 included in primary analysis
- 2.4 **No** An independent assessor not involved in treatment delivery, but no blinded, conducted all assessments. Acknowledged in study limitations and potential for bias.
- 2.5 **Yes** 88% of participants (71/80) received their allocated intervention

Paper 8 (Le Grange et al., 2015) – Criteria complete 100%: Outcome **HIGH QUALITY**

- 2.1 **Yes** One hundred thirty participants agreed to randomization (97% of eligible participants). Randomisation was done in unequal proportions of 2:2:1 for FBT-BN: CBT-A: SPT. Randomization, with no stratification on baseline variables, was done by staff at the Data Co-ordinating Centre (DCC), but separately for each site. Treatment conditions were randomly ordered before consecutive identification numbers were assigned. Families were notified of treatment assignment prior to Session 1, with participants considered entered when told their treatment allocation
- 2.2 **Yes** Commented on baseline descriptive statistics by both centre and treatment
- 2.3 **Yes** Paper reports that they included all randomized individuals (ITT principle) in the analysis if data from at least one of the four assessment was available, with missing data treated as missing at random (MAR). Study drop out reported as 10% at end of treatment, 38% at 6 month follow-up and 36% at 12 month follow-up.
- 2.4 **Yes** Commented that independent and trained assessors, who were not involved in the treatment and intended to be blind to treatment assignment, conducted all assessments. A site specific child and adolescent psychiatrist blind to participants psychological treatment managed co-morbid psychiatric conditions requiring medications. A site specific paediatrician blind to patient assignment provided oversight and determined medical instability that would warrant hospitalisation based on published criteria.
- 2.5 **Yes** Reported that 117/130 (90%) received their planned intervention

Paper 14 (Schmidt et al., 2007) – Criteria Complete 80% Outcome **MODERATE QUALITY**

- 2.1 **Yes** Paper reports "Randomization sequence to family therapy or guided self-care was generated by an independent statistician, using permutated blocks of random sizes between 4 and 1. Treatment assignment codes were contained in a computerized randomization database that concealed the sequence until interventions were assigned. Names were entered into the database by an independent administrator, and the treatment assignment was conveyed to the assessing clinician, who then informed the patient"
- 2.2 **Yes** Reported on baseline demographic and clinical characteristics of both groups, although noted that data was not available for all participants on each measure.
- 2.3 **Yes** 41/41 and 44/44 included in analysis
- 2.4 **Yes** "Those who consented to participate were assessed by a research assistant who remained blind to the treatment assignment throughout the study"
- 2.5 **No** Reports 60/85 (70%) completed intended intervention

Paper 15 (Stefini et al., 2017) – Criteria Complete 80% Outcome **MODERATE QUALITY**

- 2.1 **Yes** Paper reports "participants were randomly assigned to receive either CBT or PDT using block randomization, which was conducted by a research assistant who was not involved in the diagnostic procedures or outcome evaluation. Participants were informed about their treatment assignment after completing the baseline assessments.
- 2.2 **Yes** Paper reports "The baseline data for age, diagnosis, binge frequency (last 28 days), purge frequency (last 28 days), and EDE measures did not significantly diff between treatment groups and sites. Table reporting baseline patient characteristics by treatment group also reported.
- 2.3 **Yes** 39/39 and 42/42 included in analysis using intention to treat model
- 2.4 **Yes** Reports "Trained psychologists blinded to treatment condition administered the outcome measure at baseline, during treatment, at the end of treatment and 12 months after treatment".
- 2.5 **No** Reports 55/81 (68%) completed initial intervention

Paper 18 (Wagner et al., 2013) – Criteria Complete 0% Outcome **LOW QUALITY**

- 2.1 **Can't Tell** Paper methodology reports "one hundred and twenty-six patients with BN were randomly allocated to a cognitive behaviour therapy based self-help program delivered by the internet or bibliography, both accompanied by email guidance" hence inclusion under RCT study design. However, did not comment further on the randomisation process.
- 2.2 **Can't Tell** Whilst paper comments on group demographics for adolescents versus adults, it does not compare adolescent BN groups (INT-GSH vs BIB-GSH), reported that both treatment groups were combined due to no outcome differences between the treatment groups.
- 2.3 **Can't Tell** Doesn't comment on whether complete outcome data/whether any ITT analysis used
- 2.4 **Can't Tell** Paper methodology did not comment on blinding
- 2.5 **No** Reports "The majority of participants had participated with a minimum of 8 weeks (completers), 69% (n=20) in the adolescent group.

Secondary RCT Analysis (n =3)

	10	13	17
2.1	Y	Y	Y
2.2	Y	Y	Y
2.3	Y	Y	Y
2.4	Y	Y	Y
2.5	Y	Y	Y

Comments:

Paper 10 (Matheson et al, 2024): Criteria Complete 100% Outcome **HIGH QUALITY**

- 2.1 **Yes** *Secondary Analysis of Le Grange 2015 RCT see Paper 8 comments*
- 2.2 **Yes** *Secondary Analysis of Le Grange 2015 RCT see Paper 8 comments*
- 2.3 **Yes** *Secondary Analysis of Le Grange 2015 RCT see Paper 8 comments*
- 2.4 **Yes** *Secondary Analysis of Le Grange 2015 RCT see Paper 8 comments*
- 2.5 **Yes** *Secondary Analysis of Le Grange 2015 RCT see Paper 8 comments*

Paper 13 (Reilly et al, 2022): Criteria Complete 100% Outcome **HIGH QUALITY**

- 2.1 **Yes** *Secondary Analysis of Le Grange 2015 RCT see Paper 8 comments*

- 2.2 **Yes** *Secondary Analysis of Le Grange 2015 RCT see Paper 8 comments*
- 2.3 **Yes** *Secondary Analysis of Le Grange 2015 RCT see Paper 8 comments*
- 2.4 **Yes** *Secondary Analysis of Le Grange 2015 RCT see Paper 8 comments*
- 2.5 **Yes** *Secondary Analysis of Le Grange 2015 RCT see Paper 8 comments*

Paper 17 (Valenzuela et al, 2018): Criteria Complete 100% Outcome **HIGH QUALITY**

- 2.1 **Yes** *Secondary Analysis of Le Grange 2015 RCT see Paper 8 comments*
- 2.2 **Yes** *Secondary Analysis of Le Grange 2015 RCT see Paper 8 comments*
- 2.3 **Yes** *Secondary Analysis of Le Grange 2015 RCT see Paper 8 comments*
- 2.4 **Yes** *Secondary Analysis of Le Grange 2015 RCT see Paper 8 comments*
- 2.5 **Yes** *Secondary Analysis of Le Grange 2015 RCT see Paper 8 comments*

3. Quantitative Non-Randomized (n=0)

4. Quantitative Descriptive (n=9)

- 4.1: Is the sampling strategy relevant to address the research question?
- 4.2: Is the sample representative of the target population?
- 4.3: Are the measurements appropriate?
- 4.4: Is the risk of nonresponse bias low?
- 4.5: Is the statistical analysis appropriate to answer the research question?

Answer options: Yes (Y), No (N), Can't tell (CT)

	**1**	**4**	**5**	**6**	**9**	**11**	**12**	**16**
**4.1**	Y	Y	Y	Y	Y	Y	Y	Y
**4.2**	Y	Y	Y	Y	Y	Y	Y	Y
**4.3**	Y	Y	Y	Y	Y	Y	Y	Y
**4.4**	Y	**N**	**CT**	**N**	Y	Y	**N**	**CT**
**4.5**	CT	Y	Y	CT	Y	Y	Y	Y

Comments:

Paper 1 (Dodge et al., 1995) – Criteria Complete 80% Outcome **MODERATE QUALITY**

- 4.1 **Yes** Participants selected from 57 subsequent referrals to Eating Disorder Service of the Department of Children and Adolescents at the Maudsley Hospital between late 1988 and early 1991. 10 of which had Bulimia Nervosa.
- 4.2 **Yes** Participants were all young women between 14.5 to 17.75 years at the time of assessment, all but one met the criteria for a diagnosis of Bulimia Nervosa using DSM-III-R and all met ICD-10 criteria.
- 4.3 **Yes** Appropriate measures used: Morgan Russel Scales for Anorexia Nervosa with additional ratings added for bulimic symptoms used at assessment and follow-up, covers behaviours over the preceding 6 months (5 scales – nutritional status, menstrual function, mental state, psychosexual and socio-economic adjustment- calculate average outcome score), also used General outcome score (good- no bulimic symptoms, intermediate – bulimic symptoms <1/week) or poor outcomes – bulimic symptoms >1/week). Also completed EAT-50, EDI, RSE at baseline and reassessment.
- 4.4 **Yes** Complete outcome data available
- 4.5 **Can't Tell** Did not specifically comment on statistical analysis plan, although did report p values, t values and standard deviations.

Paper 4 (Kotler et al., 2003) – Criteria Complete 80% Outcome **MODERATE QUALITY**

- 4.1 **Yes** Source of sample is relevant to the research question; recruited from child psychiatry clinics, paediatricians, schools and community therapists. Study cohort included 10 adolescents (age 12-18 years) with bulimia nervosa. All participants had DSM-IV diagnosis of BN or EDNOS.
- 4.2 **Yes** Study focussed on 10 adolescents (age 12-18 years) with bulimia nervosa

- 4.3 **Yes** Primary outcome measure = frequency of binge eating and purging and ratings on clinical global impressions-improvement scale CGI-I), secondary outcome measures included self-report measures of eating disorder, depression, and anxiety symptoms.
- 4.4 **No** 7/13 (54%) reportedly completed intervention in total
- 4.5 **Yes** Commented on primary outcome measures being frequency of binge eating and purging, as well as eating disorder, depression, and anxiety self report measures. Treatment effects having been assessed with paired t-tests (two tailed) to assess for change from baseline to week 8 or last observation carried forward.

Paper 5 (Lazaro et al., 2011) – Criteria Complete 80% Outcome **MODERATE QUALITY**

- 4.1 **Yes** appropriate intervention (social esteem group therapy as part of a day hospital programme) subdivided Bulimia Nervosa/Bulimia Nervosa and related disorders. Appropriately sampled from EDU of Barcelona Child & Adolescent Psychiatry Hospital. Addressed Aim 2 of research question.
- 4.2 **Yes** Sample is representative of the target population (adolescent with BN)
- 4.3 **Yes** pre and post therapy PHC-SCS, SEED and Socialization Battery (BAS-3)
- 4.4 **Cant Tell** Did not comment on whether complete outcome data or drop out rate
- 4.5 **Yes** Appropriate description of statistical analysis methodology to compare pre and post intervention means of continuous variables

Paper 6 (Lebow et al., 2023) – Criteria Complete 60% Outcome **LOW QUALITY**

- 4.1 **Yes** Source of sample is relevant to the research question; recruited from local providers and online advertisements.
- 4.2 **Yes** Study cohort included 8 adolescents (age 14-20 years) with DSM V BN or subthreshold BN meeting criteria for OSFED All participants had DSM-IV diagnosis of BN or EDNOS.
- 4.3 **Yes** Pre and post intervention EDE as well as baseline and EOT self report and parental measures
- 4.4 **No** Although all participants completed the clinician-administered interviews (EDE at baseline and EOT), none of the 5 participants or parents who were given EOT self-report measures to complete at home and return via mail did so
- 4.5 **Can't Tell** Did not specifically comment on statistical analysis plan, although did report effect sizes (d), means and SD

Paper 9 (Martinez-Mallen et al., 2007) – Criteria Complete 100% Outcome **HIGH QUALITY**

- 4.1 **Yes** sampling strategy appropriate for research question, sampled from EDU
- 4.2 **Yes** sample is representative of target population 25 female patients with DSM-IV Bulimia Nervosa- mean age 16.7
- 4.3 **Yes** appropriate outcome measures at baseline, EOT and 6 month f/u. BDI, STAIC, EAT 25, EDI-2, BULIT-R
- 4.4 **Yes** commented on 3 patients dropping out during the first 3 CE sessions and being excluded from the analysis, that the age and clinical characteristics of these patients were the same as those who completed treatment. 88% participants completed treatment.
- 4.5 **Yes** Described appropriate statistical analysis to answer research question comparing pre and post intervention outcome measures

Paper 11 (Murray et al., 2015) – Criteria Complete 100% Outcome **HIGH QUALITY**

- 4.1 **Yes** Sampling strategy appropriate to research question. 40 consecutive referrals to partial hospital eating disorders treatment program, with no exclusion criteria (naturalistic)
- 4.2 **Yes** Included adolescent females with primary diagnosis of BN
- 4.3 **Yes** Appropriate Outcome measures at intake and discharge, EDE-Q, frequency of binge purge behaviours, DERSE, PVA
- 4.4 **Yes** commented on those 5 individuals whose data was not included in the study and that all patients completed measures at both intake and discharge
- 4.5 **Yes** Described appropriate statistical analysis to answer research question comparing pre and post intervention outcome measures

Paper 12 (Pretorius et al., 2009) – Criteria Complete 80% Outcome **MODERATE QUALITY**

- 4.1 **Yes** Sampling strategy appropriate to research question. Taken from consecutive referrals to one of nine specialist eating disorder clinics or from BEAT.
- 4.2 **Yes** Adolescents fulfilling DSM-IV criteria for BN or EDNOS with bulimic features
- 4.3 **Yes** Appropriate outcome measures at appropriate intervals, EDE, self report EDE-Q
- 4.4 **No** Reported 51.5% participants completed the 3 month follow-up assessment interview, 62.3% completed the 6 month assessment and 70% completed at least one follow-up assessment (3/6 month) did comment the lack of differences between those who completed and did not complete follow-up assessments.
- 4.5 **Yes** Described appropriate statistical analysis to answer research question comparing pre and post intervention outcome measures

Paper 16 (Stewart et al., 2021) – Criteria Complete 80% Outcome **MODERATE QUALITY**

- 4.1 **Yes** Sampling strategy appropriate to research question. Referrals to MFT-BN as part of their treatment for BN in a community eating disorder service.
- 4.2 **Yes** Adolescents fulfilling criteria for BN
- 4.3 **Yes** Appropriate outcome measures at appropriate intervals, EDEQ, RCADS, DERS HADS
- 4.4 **Cant Tell** No comment on loss to follow-up, or participant drop out, unable to comment on non response levels and whether there was subsequent bias.
- 4.5 **Yes** Described appropriate statistical analysis to answer research question comparing pre and post intervention outcome measures

5. Mixed methods (n=0)

References

Dodge, E., Hodes, M., Eisler, I., & Dare, C. (1995). Family therapy for bulimia nervosa in adolescents: An exploratory study. *Special Issue: Eating Disorders*, *17*(1), 59–77. https://doi.org/10.1111/j.1467-6427.1995.tb00004.x

Field, T., Schanberg, S., Kuhn, C., Fierro, K., Henteleff, T., Mueller, C., Yando, R., Shaw, S., & Burman, I. (1998). Bulimic adolescents benefit from massage therapy. *Adolescence*, *33*(131), 555–555. CINAHL.

Hong, Q. N., Pluye, P., Fabregues, S., Bartlett, G., Broardman, F., Cargo, M., Dagenais, P., Gagnon, M.-P., Griffiths, F., Nicolau, B., O'Cathain, A., Rousseau, M.-C., & Vedel, I. (2018). *Mixed Method Appraisal Tool (MMAT)*. http://mixedmethodsappraisaltoolpublic.pbworks.com/

Johnson, S. M., Maddeaux, C., & Blouin, J. (1998). Emotionally focused family therapy for bulimia: Changing attachment patterns. *Psychotherapy*, *35*(2), 238–247. https://doi.org/10.1037/h0087728

Kotler, L. A., Devlin, M. J., Davies, M., & Walsh, B. T. (2003). An Open Trial of Fluoxetine for Adolescents with Bulimia Nervosa. *Journal of Child and Adolescent Psychopharmacology*, *13*(3), 329–335. https://doi.org/10.1089/104454603322572660

Lazaro, L., Font, E., Moreno, E., Calvo, R., Vila, M., Andres-Perpina, S., Canalda, G., Martinez, E., & Castro-Fornieles, J. (2011). Effectiveness of self-esteem and social skills group therapy in adolescent eating disorder patients attending a day hospital treatment programme. *European Eating Disorders Review : The Journal of the Eating Disorders Association*, *19*(5), 398–406. https://doi.org/10.1002/erv.1054

Le Grange, D., Crosby, R. D., Rathouz, P. J., & Leventhal, B. L. (2007). A Randomized Controlled Comparison of Family-Based Treatment and Supportive Psychotherapy for Adolescent Bulimia Nervosa. *Archives of General Psychiatry*, *64*(9), 1049–1056. https://doi.org/10.1001/archpsyc.64.9.1049

Le Grange, D., Lock, J., Agras, W. S., Bryson, S. W., & Jo, B. (2015). Randomized Clinical Trial of Family-Based Treatment and Cognitive-Behavioral Therapy for Adolescent Bulimia Nervosa. *Journal of the American Academy of Child & Adolescent Psychiatry*, *54*(11), 886-894.e2. https://doi.org/10.1016/j.jaac.2015.08.008

Lebow, J., Sim, L., Wonderlich, S., & Peterson, C. B. (2023). Adapting integrative cognitive-affective therapy for adolescents with full and subthreshold bulimia nervosa: A feasibility study. *European Eating Disorders Review*, *31*(1), 178–187. https://doi.org/10.1002/erv.2946

Martinez-Mallen, E., Castro-Fornieles, J., Lazaro, L., Moreno, E., Morer, A., Font, E., Julien, J., Vila, M., & Toro, J. (2007). Cue exposure in the treatment of resistant adolescent bulimia nervosa. *International Journal of Eating Disorders*, *40*(7), 596–601. https://doi.org/10.1002/eat.20423

Murray, S. B., Anderson, L. K., Cusack, A., Nakamura, T., Rockwell, R., Griffiths, S., & Kaye, W. H. (2015). Integrating Family-Based Treatment and Dialectical Behavior Therapy for Adolescent Bulimia Nervosa: Preliminary Outcomes of an Open Pilot Trial. *Eating Disorders*, *23*(4), 336–344. https://doi.org/10.1080/10640266.2015.1044345

Pretorius, N., Treasure, J., Waller, G., Yoshioka, M., Schmidt, U., Arcelus, J., Beecham, J., Dawson, H., Morris, J., Doherty, F., Gowers, S., Rowlands, L., Eisler, I., Simic, M., Gallagher, C., Johnson-Sabine, E., Richards, L., Isaacs, G., Jones, A., … Yi, I. (2009). Cognitive-behavioural therapy for adolescents with bulimic symptomatology: The acceptability and effectiveness of internet-based delivery. *Behaviour Research and Therapy*, *47*(9), 729–736. https://doi.org/10.1016/j.brat.2009.05.006

Schmidt, U., Lee, S., Beecham, J., Perkins, S., Treasure, J., Yi, I., Winn, S., Robinson, P., Murphy, R., Keville, S., Johnson-Sabine, E., Jenkins, M., Frost, S., Dodge, L., Berelowitz, M., & Eisler, I. (2007). A Randomized Controlled Trial of Family Therapy and Cognitive Behavior Therapy Guided Self-Care for Adolescents With Bulimia Nervosa and Related Disorders. *American Journal of Psychiatry*, *164*(4), 591–598. https://doi.org/10.1176/ajp.2007.164.4.591

Stefini, A., Salzer, S., Reich, G., Horn, H., Winkelmann, K., Bents, H., Rutz, U., Frost, U., von Boetticher, A., Ruhl, U., Specht, N., & Kronmüller, K.-T. (2017). Cognitive-Behavioral and Psychodynamic Therapy in Female Adolescents With Bulimia Nervosa: A Randomized Controlled Trial. *Journal of the American Academy of Child and Adolescent Psychiatry*, *56*(4), 329–335. https://doi.org/10.1016/j.jaac.2017.01.019

Stewart, C. S., Baudinet, J., Hall, R., Fiskå, M., Pretorius, N., Voulgari, S., Hunt, K., Eisler, I., & Simic, M. (2021). Multi-family therapy for bulimia nervosa in adolescence: A pilot study in a community eating disorder service. *Eating Disorders*, *29*(4), 351–367. https://doi.org/10.1080/10640266.2019.1656461

Wagner, G., Nobis, G., Mayerhofer, A., Schau, J., Spitzer, M., Karwautz, A., Penelo, E., & Imgart, H. (2013). Is technology assisted guided self-help successful in treating female adolescents with bulimia nervosa? *Neuropsychiatrie*, *27*(2), 66–73. https://doi.org/10.1007/s40211-013-0062-x
