# Supplementary material for: Treatment outcomes for adolescent bulimia nervosa: a systematic scoping review of quantitative findings
Source: J Eat Disord. 2025 Apr 16;13:68. doi: 10.1186/s40337-025-01236-8 (PMC12004555; doi:10.1186/s40337-025-01236-8)
Supplement: Supplementary file 3 — Additional file 3. [file 40337_2025_1236_MOESM3_ESM.pdf]

Table 3: Impact of interventions on physical and psychological symptoms of adolescent bulimia nervosa (aim 1)

Author (year) [Country], Design (Setting)[a]	Mean age (SD), Sample n, % female, race/ethnicity, SES, Diagnosis	Intervention	Outcomes (Measured at)	Baseline data, mean (SD)	End-of-treatment and follow-up data, mean (SD) p, effect size
Randomised controlled trials					
Field et al (1998) [USA]<br><br>RCT (Residential)	nr (nr, range 16-21)<br><br>N = 24<br>F: 100%<br>E: Hispanic 68%, Non-Hispanic White 32%,<br>SES: Middle to upper SES [2.2 on Hollingshead Index])<br><br>DSM-III-R BN (100%)	Massage + TAU vs TAU<br><br>10 massages/5 weeks	Change in EDI mean total scores<br><br>(First Day/Last Day)	First Day<br>Massage group: 87.6 (sd nr)<br>Control: 81.2 (sd nr)	Final Day<br>Massage group: 48.7 (sd nr), $p_w = 0.001$, ES nr<br>Control: 82.7 (sd nr), p reported as "not significant", ES nr<br><br>Significant interaction effect described. Massage group had improved EDI subscale scores (drive for thinness, bulimia, body dissatisfaction, ineffectiveness, perfectionism, interpersonal distrust, interoceptive awareness, and maturity fears) compared to control group (p's nr, ES nr)<br><br>NB: Individual EDI subscale data not reported.
Johnson et al (1998) [USA]<br><br>RCT (OP)	17 (nr)<br><br>N = 13<br>F: 100%<br>E: nr<br>SES: nr)<br><br>DSM-III-R BN (100%)	EFFT vs group CBT<br><br>10 weeks CBT Group Therapy vs 10 sessions of EFFT	Changes in the following measures:<br>DSES<br>EDI<br>BSCL<br><br>(not reported)	Not reported	MANOVAs showed both EFFT and CBT group experienced a decrease in bulimic symptoms as per improved EDI scores ($p \leq 0.05$, ES nr) and BSCL scores ($p \leq 0.05$, ES nr). No between group effects (p nr, ES nr).<br><br>EFFT subgroup:<br>52% binge frequency reduction with 44% complete remission. 65% purge frequency reduction with 66.6% complete remission. The following EDI subscales also reduced significantly: bulimia ($p < 0.05$, "effect size" = 1.1) drive for thinness ($p < 0.01$, ES nr), and ineffectiveness ($p < 0.01$, ES nr). Using paired t tests (pre and post) a significant reduction was noted on the BSCL self-rating scores of bingeing severity ($p < 0.01$, "effect size" = 0.87) and vomiting severity ($p < 0.01$, ES nr).
Le Grange et al (2007) [USA]<br><br>RCT (OP)	16.1 (1.7)<br><br>N = 80<br>F: 98%<br>E: White 64%, Hispanic 20%, African American 11%, Other 5%<br>SES: nr<br><br>DSM-IV BN (46.2%)<br>Subthreshold BN (53.8%)	FBT-BN (n = 41) vs SPT (n = 39)<br><br>20 sessions/6 months	Primary<br>Proportion of participants remitted or partially remitted, as per EDE<br><br>(EOT, 6mfu)<br><br>Secondary<br>Behavioural: OBE, SBE, vomiting, all compensatory behaviours	Primary<br>n/a<br><br>Secondary<br>FBT-BN:<br>Behavioural:<br>OBE: 18.4 (28.1),<br>SBE: 9.9 (16.6),<br>Vomiting: 34.5 (31.0),<br>All compensatory behaviours: 49.5 (36.9)<br>EDE:<br>Restraint 3.8 (1.3)<br>Weight concern: 3.7 (1.4)	Primary<br>EOT<br>remission: FBT-BN: 39%, SPT: 18%, p = 0.049, ES nr<br>partial remission: FBT-BN: 41%, SPT: 21%, p = 0.06, ES nr<br><br>6mfu<br>remission: FBT-BN: 29%, SPT: 10%, p = 0.05, ES nr<br>partial remission: No significant difference, p nr, ES nr<br><br>Remission rates higher for FBT-BN. FBT-BN maintained this advantage over SPT at 6 months, albeit by a narrower margin. Partial remission rates at EOT were higher for FBT-BN although only approached significance.<br><br>Secondary

			EDE: subscales and global score (EDE-Q: mid-treatment only)<br><br>(Baseline, mid-treatment, EOT, 6mfu)	Shape concern: 4.0 (1.4)<br>Eating concern: 2.9 (1.4)<br>Global: 3.6 (1.1)<br><br><br>SPT:<br>Behavioural:<br>OBE 18.9 (22.3)<br>SBE 7.6 (10.1)<br>Vomiting 33.2 (33.5)<br>All compensatory behaviour 50.2 (42.3)<br>EDE:<br>Restraint 3.7 (1.7)<br>Weight concern 4.1 (1.3)<br>Shape concern 4.2 (1.1)<br>Eating concern 2.9 (1.2)<br>Global 3.7 (1.1)	EOT<br>FBT-BN: Behavioural: OBE: 4.1 (14.8), SBE: 4.5 (13.3), vomiting: 4.8 (9.4), all compensatory behaviours: 6.9 (10.2). EDE: restraint: 1.3 (1.5), weight concern: 1.8 (1.6), shape concern: 1.8 (1.6), eating concern: 1.0 (1.5), global: 1.5 (1.4)<br>SPT: Behavioural: OBE: 3.2 (5.1), SBE: 4.6 (8.6), vomiting: 17.4 (26.0), all compensatory behaviours: 22.3 (28.6). EDE: restraint: 2.1 (1.6), weight concern: 2.6 (1.7), shape concern: 2.7 (1.7), eating concern: 1.5 (1.4), global: 2.2 (1.4)<br><br>By EOT, FBT-BN demonstrated significantly greater reductions in vomiting (p nr, "effect size" = 0.62), all compensatory behaviours ("effect size" = 0.68), and the restraint subscale on the EDE (p nr, "effect size" = 0.50). No significant change on other indices (p nr, ES, nr)<br><br>6mfu<br>FBT-BN: Behavioural: OBE: 2.5 (6.8), SBE: 2.8 (6.9), vomiting: 10.1 (21.8), all compensatory behaviours 12.4 (21.6) EDE: restraint 1.3 (1.5), weight concern 1.6 (1.5), shape concern 1.7 (1.5), eating concern 0.8 (1.2), global 1.4 (1.2)<br>SPT: Behavioural: OBE: 5.4 (13.7), SBE: 2.4 (5.2), vomiting: 14.5 (27.7), all compensatory behaviours: 17.9 (28.0). EDE: restraint: 1.9 (1.6), weight concern: 2.3 (1.5), shape concern: 2.7 (1.9), eating concern: 1.3 (1.5), global: 1.9 (1.4)<br><br>At 6mfu, no statistically significant difference between the two groups (p's nr, ES nr).
Le Grange et al (2015) [USA]<br><br>RCT (OP)	15.8 (1.5)<br><br>N = 109<br>F: 94%<br>E: 46% "ethnic minority"<br>SES: Income >100K (CBT-A 40%, FBT-BN 37%), Parent with college degree: [CBT-A 74%, FBT-BN 65%])<br><br>DSM -IV BN/Partial BN (100%)	FBT-BN (n = 51) vs CBT (n = 58) vs SPT (n = 20; not included in analysis)<br><br>18 sessions/6 months	Abstinence from bingeing and purging at EOT as per EDE.<br><br>(Baseline, EOT, 6mfu, 12mfu)	Median binge eating episodes: 17.0 (IQR: 26.75)<br>Median purge episodes: 29.0 (IQR 31.5)	EOT Abstinence<br>FBT-BN: 39.4%, CBT-A: 19.7%<br>Significant between group difference in abstinence rate (mean difference: 19.7%, p = 0.040, NNT = 5)<br><br>6mfu Abstinence<br>FBT-BN: 44%, CBT-A: 25.4%<br>Significant between group difference in abstinence rate (mean difference: 18.5%, p = 0.030, NNT = 5)<br><br>12mfu Abstinence<br>FBT-BN: 49%, CBT-A: 32%<br>No between group difference in abstinence rate (mean difference: 16.5%, p = 0.130, NNT = 6)
Schmidt et al (2007) [UK]<br><br>RCT<br>(OP)	FT-BN: 17.9 (1.6)<br>CBT-GSH: 17.4 (1.8)<br><br>N = 85<br>F: 97.6%,<br>E: White 71.7%, Other Ethnicity 6%<br>SES: nr<br><br>BN: 71.7%<br>EDNOS: 28.3%	FT-BN (n = 41) vs CBT-GSH (n = 44)<br><br>FT-BN: 15 sessions/6 months<br><br>CBT-GSH: 10 weekly sessions	Primary:<br>Objective abstinence from bingeing and vomiting at EOT and 6mfu<br><br>Secondary<br>EATATE<br>SEED<br><br>(Baseline, EOT, 6mfu)	FBT-BN Group<br>Abstinence: Binging 19.5%, vomiting 14.6%, combined 5%<br>Subclinical: Binging 14.6%, vomiting 4.9%, combined 14.6%<br>Clinical: Binging 65.9%, vomiting 80.5%, combined 80.4%<br><br>CBT-GSH Group<br>Abstinence: Binging 18%, vomiting 20.5%, combined 4.5%	FT-BN Group EOT<br>Abstinence: Binging 25%, vomiting 28%, combined 12.5%<br>Subclinical: Binging 25%, vomiting 31.3%, combined 34.4%<br>Clinical: Binging 50%, vomiting 40.6%, combined 53.1%<br><br>CBT-GSH Group EOT<br>Abstinence: Binging 41.9 %, vomiting 32.3%, combined 19.4%<br>Subclinical: Binging 38.7%, vomiting 35.5%, combined 38.7%<br>Clinical: Binging 19.4%, vomiting 32.3%, combined 41.9%

				Subclinical: Binging 18.0%, vomiting 9.1%, combined 9.1%<br>Clinical: Binging 63.6%, vomiting 70.5%, combined 86.4%	On the EATATE, significant improvements in bingeing ($p < 0.0001$, ES nr) and vomiting ($p < 0.02$, ES nr) in both treatment groups over time. Higher proportion of CBT-GSH patients abstinent from self-reported bingeing at EOT compared to FBT-BN at EOT ($p = 0.03$, d = –0.21). Significant group by time interaction also showed CBT-GSH had earlier improvement than FBT-BN ($p < 0.0001$, ES nr). No between group difference in vomiting or combined bingeing and vomiting at EOT (p nr, ES nr).<br><br>FT-BN Group (6mfu)<br>Abstinence: Binging 55.0%, vomiting 51.7%, combined 41.4%<br>Subclinical: Binging 27.6%, vomiting 24.1%, combined 31%<br>Clinical: Binging 17.2%, vomiting 24%, combined 27.6%<br><br>CBT-GSH Group (6mfu)<br>Abstinence: Binging 52%, vomiting 56%, combined 36.0%<br>Subclinical: Binging 20.0%, vomiting 12.0%, combined 24%<br>Clinical: Binging 28%, vomiting 32%, combined 40%<br><br>No between group difference in bingeing, vomiting or combined bingeing/vomiting at 6mfu (p nr, ES nr).
Stefini et al (2017) [Germany]<br><br>RCT<br>(OP)	18.7 (1.9)<br><br>N = 81<br>F: 100%<br>E: nr<br>SES: nr<br><br>DSM-IV BN (77.7%), DSM-IV Partial BN (22.3%)	CBT (n = 39) vs PDT (n = 42)<br><br>Up to 60 sessions/1yr	Primary<br>Remission, defined as a lack of DSM-IV diagnosis for BN or partial BN by EOT<br><br>Secondary<br>EDE, EDE-Q Objective binges/28 days, purging episodes/28 days.<br><br>(Baseline, during treatment, EOT, 12mfu)	Primary<br>Diagnosis (Yes/No):<br>CBT Group: 39/39 (100%/0%)<br>PDT Group: 42/42 (100%/0%)	Primary (EOT)<br>Diagnosis (Y/N):<br>CBT: 26/13 (66.7%/33.3%) p_w nr, h = 1.22<br>PDT: 30/12 (69.8%/30.2%), p_w nr, h = 1.18<br><br>No significant between group difference observed ($p_b = 0.82$, h = 0.05; OR = 0.90 [95%CI: 0.35-2.28])<br><br>Secondary (EOT)<br>CBT: Frequency of binge ($p_w < 0.001$, d = 0.61) and purge ($p_w < 0.001$, d = 0.66) behaviour decreased significantly from baseline to EOT. Significant improvement on all EDE and EDE-Q scales (all p_w's ≤ 0.001, d's = 1.41 – 1.91)<br>PDT: Significant decrease in frequency of binge eating ($p_w = 0.01$, d = 0.44) and purging ($p_w = 0.05$, d = 0.31). Significant improvement on all EDE and EDE-Q scales (all $p_w \leq 0.001$, d's = 1.39 – 1.82).<br><br>No significant between group difference observed (all p_b's > 0.05, d's = 0.01 – 0.35)<br><br>12mfu (24 months post-baseline)<br>Rates of remission stable from EOT to 12mfu (CBT: $p = 0.42$, ES nr; PDT: $p = 0.69$, ES nr). No significant differences between groups ($p = 0.48$, ES nr).<br><br>No significant changes from EOT to 12mfu in bingeing ($p = 0.18$, ES nr) or purging ($p = 0.16$, ES nr). No significant interaction effects for time and treatment group for bingeing ($p = 0.51$, ES nr) or purging ($p = 0.22$, ES nr).

Wagner et al (2013)<br>[Austria / Germany]<br><br>RCT (OP)	19.31 (1.77)<br><br>Adolescent BN Group:<br>N = 29<br>F: 100%<br>E: nr<br>SES: nr<br><br>DSM-IV-R BN (100%)<br><br>NB: From mixed age BN sample (97 adults, 29 adolescents [age range 16-21])	INT-GSH or BIB-GSH<br><br>4-7 months	QATA<br>EDI-2<br><br>(Baseline, 4,7 and 18 months)	OBE: 46.31 (57.29)<br>Vomiting: 73.44 (104.2)<br>Excessive sport: 3.94 (8.00)<br>Fasting: 11.13 (20.42)<br>Laxative abuse: 0.38 (1.02)<br><br>EDI-2 total score: 82.19 (42.71)	Month 4<br>OBE: 30.88 (40.14), vomiting: 29.88 (41.26), excessive sport: 2.75 (5.00)<br>fasting: 1.00 (2.30), laxative abuse: 0.06 (0.25), EDI-2 total score: 55.50 (45.30)<br><br>Month 7 (EOT)<br>OBE: 31.00 (40.14), vomiting: 30.25 (59.17), excessive sport: 5.25 (13.99)<br>Fasting: 5.00 (14.39), laxative abuse: 0.56 (2.25), EDI-2 total score: 49.53 (42.62)<br>Remission or abstinent y/n: (8/18) 44.4% vs 38.7% adults (p = 0.662)<br><br>Month 18<br>OBE: 17.88 (29.20), vomiting: 18.19 (29.61), excessive sport: 3.06 (5.90), fasting 1.93 (7.22), laxative abuse 0.06 (0.25), EDI-2 total score: 50.93 (36.08)<br>Remission or abstinent y/n: (11/20) 55.0% vs 62.5% adults (p = 0.556)<br><br>OBE (p < 0.001, ES nr) and vomiting (p < 0.001, ES nr) improved significantly over time, with the highest decrease during the first 4 months. All EDI-2 subscales and total score significantly improved over treatment (all p's = <0.001 – 0.031, ES nr)

RCT secondary analysis

Matheson et al (2024)<br>[USA]<br><br>Secondary Analysis (Le Grange RCT 2015)<br><br>(OP)	15.94 (1.53)<br><br>N = 51<br>F: 92%<br>E: Hispanic 23.5%, Caucasian 76.5%<br>SES: Income >100K 37%, Parent with college degree 65%<br><br>DSM -IV BN/Partial BN (100%)	FBT-BN<br><br>18 sessions/ 6 months	LOC eating episodes as per EDE. LOC episodes calculated as sum of objective and subjective bulimic episodes over the previous month.<br><br>(Baseline, EOT, 6mfu, 12mfu)	Baseline LOC episodes<br>Mean: 25 (25.60)<br>Median: 17 (range: 1-134)	EOT LOC episodes<br>Mean: 4.07 (7.43), p <.001, d = 0.86<br>Median: 0 (range: 0-28)<br><br>Large effect size change from baseline to EOT.<br><br>6mfu LOC episodes<br>Mean: 7.54 (16.84), p > .05, d = -0.26<br>Median: 0 (range: 0-84)<br><br>Small effect size increase in episodes between EOT and 6mfu.<br><br>12mfu LOC episodes<br>Mean: 6.7 (18.98), p = .003, d = 0.53<br>Median: 0 (range: 0-86)<br><br>Medium effect size decrease in LOC from pre-treatment to 12mfu (p = .003, d = 0.53).<br>Small effect size from 6mfu to 12mfu (p > .05, d = 0.07).

Single-arm studies

Dodge et al (1995) [UK]<br><br>Case Series (OP)	16.5 (1.21)<br><br>N = 8<br>F: 100%	FT-BN<br><br>Range: 1-16 sessions/1-17 months	Morgan Russell Scale<br>EAT-40<br>EDI<br><br>(Baseline, 12 months)	Binging: 8.3 (nr)<br>Vomiting: 8.6 (nr)<br>Laxatives: 4.9 (nr)	Binging: 5.6 (nr), p = 0.05, ES nr<br>Vomiting: 3 (nr), p = 0.05, ES nr<br>Laxatives: 2.6 (nr), p = 0.05, ES nr<br><br>Morgan Russell Average Outcome Scores 8.90 (2.32)

	E: Asian/Afro Caribbean 12.5%, White British 87.5%<br>SES: nr<br><br>ICD-10 BN (100%)			Morgan Russell Average Outcome Scores: 8.34 (2.01)<br>Socioeconomic adjustment subscale: 9.2 (nr)<br>All other subscales: nr<br>EAT: 78.38 (40.75)<br>EDI: nr	Socioeconomic adjustment subscale reported: 10.9 (nr), p = 0.03, ES nr<br>All other subscales: nr<br>Morgan Russell Outcome: good: 1/8, intermediate: 5/8, poor: 2/8<br>EAT: 57.71 (36.39), p = 0.045, ES nr<br>EDI: 'decreased', p nr, ES nr<br><br>Significant improvement in BN behaviours and eating attitudes, although EDI/EAT scores suggest significant disturbance of eating attitudes at EOT.
Kotler et al (2003) [USA]<br><br>Case Series (OP)	16.2 (1.0)<br><br>N = 10<br>F: 100%<br>E: Caucasian 50%, Hispanic 30%, Asian American 10%, Indian 10%,<br>SES: nr<br><br>DSM-IV BN (80%)<br>EDNOS (20%)	Fluoxetine 60mg/day with supportive psychotherapy<br><br>8 weeks	Primary outcome:<br>CGI improvement in frequency of binge-purge (food diaries)<br><br>(Baseline, weekly, EOT)<br><br>Secondary Outcome:<br>EDI, EAT, BSQ<br><br>(Baseline, monthly, EOT)	Primary Outcome<br>Average weekly binges: 4.1 (3.8)<br>Average weekly purges: 6.5 (5.1)<br><br>Secondary Outcome<br>EAT: 56.5 (27.2)<br>EDI (bulimia subscale): 10.6 (6.8)<br>BSQ: 151.1 (36.5)	Primary Outcome<br>Average weekly binges: 0 (0), p < 0.01, ES nr<br>Average weekly purges: 0.4 (0.9), p < 0.005, ES nr<br><br>CGI Improvement: Much improved 20%, Improved 50%, Slightly improved 30%<br><br>Secondary Outcome<br>EAT: 34.5 (20.1), p < 0.05, ES nr<br>EDI (Bulimia subscale): 4.2 (3.9), p < 0.01, ES nr<br>BSQ: 131 (41.3), p "not significant", ES nr<br><br>Average weekly binges and purges decreased significantly. All patients showed some improvement on the CGI scale. Significant improvement on both EAT and EDI bulimia subscales.
Lebow et al (2022) [USA]<br><br>Case Series (OP)	16.1 (sd nr)<br><br>N = 8<br>F: 75%,<br>E: "White" 88%<br>SES: nr<br><br>DSM-V BN (63%)<br>DSM-V OSFED BN (37%)	ICAT-A<br><br>10-27 sessions	EDE<br><br>(Baseline, EOT)	Global: 3.18 (1.38)<br>Shape concern: 3.75 (1.62)<br>Weight concern: 3.33 (1.74)<br>Eating concern: 2.43 (1.27)<br>Restraint: 3.23 (1.41)<br><br>OBE: 9.75 (15.12)<br>SBE: 17.88 (21.15)<br>Compensatory behaviours: 11.39 (9.21)	Global: 0.43 (0.40), p nr, d = 2.71<br>Shape concerns: 0.98 (0.87), p nr, d = 2.13<br>Weight concerns: 0.47 (0.53), p nr, d = 2.22<br>Eating concerns: 0.13 (0.21), p nr, d = 2.53<br>Restraint: 0.13 (0.24), d = p nr, 3.07<br><br>OBE: 0 (0), p nr, d = 0.91<br>SBE: 0.17 (0.40), p nr, d = 1.18<br>Compensatory behaviours: 0.17 (0.41), p nr, d = 1.72<br><br>Moderate to large effect of ICAT-A on all EDE subscales, binge episodes and compensatory behaviours. All completers met criteria for eating disorder remission at EOT (EDE below clinical cut-off)
Martinez-Mallen et al (2007) [Spain]<br><br>Case Series (OP/Day Hospital)	16.7 (SD 1.5)<br><br>N = 25<br>F: 100%<br>E: nr<br>SES: nr<br><br>DSM-IV BN (100%) (not responded to standard care)	Cue Exposure Program<br><br>12 session/6-weeks	EAT-26<br>EDI-2<br>BULIT-R<br><br>(Baseline, EOT, 6mfu)	Binge/week: 2.90 (3.26)<br>Vomits/week: 3.20 (5.26)<br>BULIT-R: 119.76 (3.28)<br>EDI-2 bulimic factor: 10.30 (1.43)<br>EAT-26: 39.15 (16.13)	EOT<br>Binge/week: 1.35 (2.48)<br>Vomits/week: 3.05 (6.64)<br>BULIT-R: 71.69 (7.86)<br>EDI-2 bulimic factor: 2.84 (1.15)<br>EAT-26: 18.53 (17.19)<br><br>6mfu<br>Binge/week: 0.2 (0.52), p = 0.005, ES nr<br>Vomits/week: 1.4 (3.15), p = 0.439, ES nr<br>BULIT-R: 66.38 (8.06), p < 0.001, ES nr<br>EDI-2 bulimic factor: 1.69 (0.55), p < 0.001, ES nr

					EAT-26: 19.69 (18.33), p = 0.018, ES nr<br>NB: p values from one-way ANOVA using 3 timepoints (baseline, EOT, 6m FU)<br><br>Significant improvement in binge episodes, eating attitudes (EAT-26) and bulimic symptoms (BULIT and EDI-bulimia factor), sustained at 6mfu. No significant reduction in vomiting.
Murray et al (2015) [USA]<br><br>Case Series (PHP)	15.7 (1.11)<br><br>N = 35<br>F: 100%,<br>E: Caucasian 63.8%, Hispanic 14.5%, Asian 2.9%, Black 2.9%, Other 15.9%<br>SES: nr<br><br>BN (100%)	Integrated FBT and DBT<br><br>3-10hrs/day, up to 6days/week. Mean treatment length 77.18 days (SD 38.91)	EDE-Q<br><br>(Baseline, EOT)	Restraint: 3.52 (1.86)<br>Eating concerns: 3.69 (1.26)<br>Shape concerns: 4.97 (1.47)<br>Weight concerns: 4.54 (1.41)<br>Global: 4.18 (1.33)<br><br>OBE: 4.03 (6.69)<br>Secretive eating: 1.24 (1.57)<br>Purge: 10.82 (11.57)	Restraint: 1.33 (1.82), p = 0.95, ES nr<br>Eating concerns: 1.69 (1.51) p = 0.47, ES nr<br>Shape concerns: 2.96 (1.97) p = 0.001, ES nr<br>Weight concerns: 2.62 (1.87) p = 0.001, ES nr<br>Global: 2.15 (1.67) p = 0.002, ES nr<br><br>OBE: 1.43 (3.66) p = 0.04, ES nr<br>Secretive eating: 0.60 (.847) p = 0.04, ES nr<br>Purge: 3.51 (2.26) p= 0.005, ES nr<br><br>Significant improvements in EDE-Q shape concerns, weight concerns and global EDE-Q scores. Significant reductions in frequency of secret eating, objective bingeing and self-induced vomiting. No significant change in EDE-Q restraint or eating concerns.
Pretorius et al (2009) [UK]<br><br>Case Series (OP)	18.8 (1.6)<br><br>N = 101<br>F: 97%<br>E: Caucasian 88%, Other / mixed ethnicity 8.9%, Ethnicity not reported 3.9%<br>SES: nr<br><br>DSM-IV BN (60.4%)<br>EDNOS-BN (39.6%)	Web-based CBT<br><br>8 sessions	EDE<br>EDE-Q<br><br>(Baseline, 3mfu, 6mfu)	Objective binge: 22.2 (2.0)<br>Vomit episodes: 34.1 (4.1)<br>Laxative episodes: 3.4 (1.3)<br>EDE global: 3.9 (0.1)	3 months<br>Objective binge: 12.4 (2.6)^<br>Vomit episodes: 19.2 (4.4)^<br>Laxative episodes: 2.7 (1.2)<br>EDE global: 2.9 (0.2)^<br>^Binge, vomit, EDE global p's range from 0.001 – 0.003, specific numbers not provided. p not reported for laxative episodes.<br><br>6 months<br>Objective binge: 12.7 (2.4), p = 0.001, ES nr<br>Vomit episodes: 19.0 (3.3), p = 0.002, ES nr<br>Laxative episodes 1.1 (0.5), p = 0.014, ES nr<br>EDE global 3.1 (0.20), p = 0.001, ES nr<br>NB: p values from linear model using 3 timepoints (baseline, 3m, 6m)<br><br>Abstinent from, or in the subclinical range for, bingeing, vomiting and laxatives: Baseline: 9/101 (9%), 3 months: 25/101 (25%), 6 months: 29/101 (29%)
Stewart et al (2021) [UK]<br><br>Retrospective audit (OP)	15.6 (1.4)<br><br>N = 50<br>F: 98%<br>E: nr<br>SES: nr<br><br>BN (100%)	MFT-BN<br><br>Weekly/4 months	EDE-Q (restraint, eating concern, shape concerns, weight concern, binge and compensatory behaviour frequency)<br><br>(Baseline, EOT)	Restraint: 3.57 (1.27)<br>Eating concern: 4.15 (1.07)<br>Shape concern: 5.27 (0.98)<br>Weight concern: 4.71 (1.22)<br><br>Median (range):<br>Bingeing episodes: 7 (0-195)<br>Purging episodes: 10 (0-195)<br>Laxative use: 0 (0-30)	Restraint: 3.00 (1.92) p = 0.035, r = -0.28<br>Eating concern: 2.89 (1.71) p < 0.001, r = -0.47<br>Shape concern: 4.29 (1.80) p < 0.001, r = -0.47<br>Weight concern: 3.64 (1.91) p < 0.001, r = -0.42<br><br>Median (range):<br>Bingeing episodes: 5 (0-100) p = 0.010, r = -0.35<br>Purging episodes: 3 (0-120) p = 0.034, r = -0.29<br>Laxative use: 0 (0-28) p = 0.171, r = -0.18

	Over exercise: 7 (0-52)	Over exercise: 2 (0-28) p = 0.520, r = -0.09<br><br>Significant reduction in self-reported ED symptoms, including shape, weight and eating concern as well as reduction in frequency of binge and purge episodes. No change in laxative use or over-exercise.

[a] *Three of the total number of studies identified in this review are not presented in this table as they did not report on BN outcomes, rather they report on co-morbid and/or parent/carer outcomes; two RCT secondary analysis (Reilly et al., 2022; Valenzuela et al., 2018) and one single-arm studies (Lazaro et al., 2010).*

Abbreviations: 6mfu, 6-month follow-up; 12mfu, 12-month follow-up; BDI, Beck Depression Inventory; BIB-GSH, bibliography-based CBT guided self-help; BN, bulimia nervosa; BSCL; Bulimic Symptom Checklist; BSQ; Body Shape Questionnaire; BULIT-R, Bulimia Test Revised; CBT, cognitive behavioural therapy; CBT-A, adolescent adapted CBT; CBT-GSH, guided self-help CBT; CGI, Clinical Global Impression scale; DSES, Diagnostic Survey for Eating Disorders; DSM, Diagnostic and Statistical Manual; EAT-26, Eating Attitudes Test; EATATE, a semi-structured interview (Anderluh et al. 2000, unpublished manuscript) based on the Longitudinal Interval Follow-Up Evaluation that includes variables from the EDE; EDE, Eating Disorder Examination; EDE-Q, Eating Disorder Examination Questionnaire; EDI, Eating Disorder Inventory; EDNOS, eating disorder not otherwise specified; EOT, end of treatment); EFFT, emotionally focused family therapy; E, ethnic minority; FBT-BN, family-based treatment for bulimia nervosa; FT-BN, family therapy or bulimia nervosa; ICAT-A, integrative cognitive affective therapy for adolescents; INT-GSH, internet-based CBT guided self-help; LOC, Loss of control; MFT-BN, multi-family therapy for bulimia nervosa; NNT, number needed to treat; nr, not reported; OBE, objective binge eating/episode; OP, outpatient; OSFED, other specified feed and eating disorder; PDT, psychodynamic therapy; PHP, partial hospitalisation program; QATA, Questionnaire Anamnesetique pour les Troubles Alimentaire; RCT, Randomised Controlled Trial; SBE, subjective binge eating/episode; SEED, self-report abstinence from bingeing and vomiting; SPT, supportive psychotherapy; SES, socioeconomic status; UK, United Kingdom; US, United States of America
